# Syndecan-4^–/–^ Mice Have Smaller Muscle Fibers, Increased Akt/mTOR/S6K1 and Notch/HES-1 Pathways, and Alterations in Extracellular Matrix Components

**DOI:** 10.3389/fcell.2020.00730

**Published:** 2020-07-31

**Authors:** Sissel Beate Rønning, Cathrine Rein Carlson, Jan Magnus Aronsen, Addolorata Pisconti, Vibeke Høst, Marianne Lunde, Kristian Hovde Liland, Ivar Sjaastad, Svein Olav Kolset, Geir Christensen, Mona Elisabeth Pedersen

**Affiliations:** ^1^Nofima AS, Ås, Norway; ^2^Institute for Experimental Medical Research, Oslo University Hospital and University of Oslo, Oslo, Norway; ^3^Bjørknes College, Oslo, Norway; ^4^Department of Biochemistry and Cell Biology, Stony Brook University, Stony Brook, NY, United States; ^5^Faculty of Sciences and Technology, Norwegian University of Life Sciences, Ås, Norway; ^6^K.G. Jebsen Center for Cardiac Research, University of Oslo, Oslo, Norway; ^7^Department of Nutrition, Institute of Basic Medical Sciences, University of Oslo, Oslo, Norway

**Keywords:** skeletal muscle, syndecan, exercise, myogenesis, Notch, decorin, Akt, ECM

## Abstract

**Background:**

Extracellular matrix (ECM) remodeling is essential for skeletal muscle development and adaption in response to environmental cues such as exercise and injury. The cell surface proteoglycan syndecan-4 has been reported to be essential for muscle differentiation, but few molecular mechanisms are known. Syndecan-4^–/–^ mice are unable to regenerate damaged muscle, and display deficient satellite cell activation, proliferation, and differentiation. A reduced myofiber basal lamina has also been reported in syndecan-4^–/–^ muscle, indicating possible defects in ECM production. To get a better understanding of the underlying molecular mechanisms, we have here investigated the effects of syndecan-4 genetic ablation on molecules involved in ECM remodeling and muscle growth, both under steady state conditions and in response to exercise.

**Methods:**

Tibialis anterior (TA) muscles from sedentary and exercised syndecan-4^–/–^ and WT mice were analyzed by immunohistochemistry, real-time PCR and western blotting.

**Results:**

Compared to WT, we found that syndecan-4^–/–^ mice had reduced body weight, reduced muscle weight, muscle fibers with a smaller cross-sectional area, and reduced expression of myogenic regulatory transcription factors. Sedentary syndecan-4^–/–^ had also increased mRNA levels of syndecan-2, decorin, collagens, fibromodulin, biglycan, and LOX. Some of these latter ECM components were reduced at protein level, suggesting them to be more susceptible to degradation or less efficiently translated when syndecan-4 is absent. At the protein level, TRPC7 was reduced, whereas activation of the Akt/mTOR/S6K1 and Notch/HES-1 pathways were increased. Finally, although exercise induced upregulation of several of these components in WT, a further upregulation of these molecules was not observed in exercised syndecan-4^–/–^ mice.

**Conclusion:**

Altogether our data suggest an important role of syndecan-4 in muscle development.

## Introduction

Skeletal muscle is a highly dynamic tissue, responding to physiological stimuli during development and exercise. It is composed of muscle cells (myofibers) collected in bundles. Both the individual myofibers and the myofiber bundles are surrounded by extracellular matrix (ECM). It was demonstrated more than 50 years ago that the satellite cells, the skeletal muscle stem cells (MuSCs), which are located between the basal lamina and sarcolemma (plasma membrane) of the skeletal muscle fibers and normally quiescent in adult muscle, become activated upon exercise, injury or disease, and are involved in the skeletal muscle regeneration (Mauro, 1961). More recently, several reports have identified MuSCs as the primary contributors to the postnatal growth, maintenance and regeneration of skeletal muscles. These cells have a remarkable ability to self-renew, expand, or undergo myogenic differentiation to fuse and restore damaged muscle (Tsivitse, 2010). The activation, proliferation and differentiation of MuSCs in adult muscle are mainly controlled by myogenic regulatory transcription factors (MRFs) such as myoblast determination protein (MyoD), myogenin, myogenic factor 5 (myf5) and 6 (myf6/mrf4). MuSCs express MyoD when they are activated and start to proliferate (Zammit et al., 2004), whereas myogenin is crucial for later stages of myogenic differentiation (Sabourin and Rudnicki, 2000).

The ECM, a complex network of collagens, in addition to glycoproteins and proteoglycans, is the niche of MuSCs and the myofibers. The small leucine rich proteoglycans (SLRPs) decorin, fibromodulin and biglycan, regulate collagen fibrillogenesis and crosslinking (Kalamajski and Oldberg, 2010; Kalamajski et al., 2014; Christensen et al., 2018). The ECM composition is highly dynamic and promotes skeletal muscle adaption in response to environmental forces during, e.g., exercise and regeneration, and during muscle growth and development. The ECM network also builds a scaffold for muscle cells to adhere, grow and differentiate, and functions as a storage and presenter of relevant muscle tissue growth factors and different cytokines during development (Thomas et al., 2015).

Previous work has also identified syndecans to be important for muscle development, maintenance and regeneration (Pisconti et al., 2012, 2016; Pawlikowski et al., 2017; Velleman and Song, 2017). The syndecans belong to a group of transmembrane proteoglycans, which consists of four members in mammals (Velleman et al., 2012). The syndecans are characterized by a large diverse extracellular domain with glycosaminoglycan (GAG) attachment sites, a conserved transmembrane domain and a short cytoplasmic domain with a unique variable domain differing between each syndecan. Syndecans have a wide spectrum of biological functions, and regulate calcium ion channels, polarization of epithelial cells, cell adhesions, and migration (Couchman et al., 2015). They also function as co-receptors of various growth factors and transduce signals into the cell through their cytoplasmic domains (Couchman, 2010). All four syndecans are expressed in developing muscles (Cornelison et al., 2001; Olguin and Brandan, 2001) and in proliferating myoblasts, but their expression is progressively lost during myogenesis (Brandan and Gutierrez, 2013). Syndecan-2 is reported to be highly expressed in early-differentiated myoblasts (Do et al., 2015). Syndecan-1 is not detected in postnatal muscle, while syndecan-3 and syndecan-4 are present and restricted to MuSCs and vascular cells (Cornelison et al., 2001; Cornelison et al., 2004). Syndecan-4 as well as syndecan-3 have roles in development and regeneration. Both are highly expressed in myoblasts and around early embryonal myotubes but are reduced around myotubes postnatally and restricted to MuSCs in young adults (Cornelison et al., 2001). Syndecan-3 regulates muscle progenitor cell homeostasis by promoting MuSC self-renewal. Syndecan-3^–/–^ mice show improved muscle regeneration upon repeated muscle injuries, and reduced muscle pathology in dystrophic mice (Pisconti et al., 2016). Syndecan-4^–/–^ mice are unable to regenerate damaged muscle and display deficient MuSC activation, proliferation, myoD expression and differentiation (Cornelison et al., 2004). Interestingly, a reduced myofiber basal lamina has also been reported in syndecan-4^–/–^ muscle (Cornelison et al., 2004), indicating possible defects in ECM biosynthesis and turnover.

Limited knowledge on molecular mechanisms of syndecan-4 in skeletal muscle exist. To get a better understanding of the role of syndecan-4 in skeletal muscle and the underlying molecular mechanisms, we have here investigated the effects of syndecan-4 genetic ablation in tibialis anterior (TA), and analyzed molecules involved in ECM remodeling and muscle growth, both under steady state conditions and in response to exercise. Reduced muscle growth, changes in multiple signaling molecules and ECM components were found when syndecan-4 was absent. These molecular changes were mostly not affected further by exercise.

## Materials and Methods

### Animal Experiments

All animal experiments were performed in accordance with the National Regulation on Animal Experimentation in accordance with an approved protocol (ID#2845 and 7696) and the Norwegian Animal Welfare Act and conform the NIH guidelines (2011). Female syndecan-4^–/–^ mice (KO) (Echtermeyer et al., 2001) were compared to either C57Bl/6j mice or syndecan-4^+/+^ bred from the same genetic background to study syndecan-4 dependent effects. Up to six mice per cage were housed in a temperature-regulated room with a 12:12-h light dark cycle, and access to food and water *ad libitum*. Animals were sacrificed by cardiac excision in deep surgical anesthesia.

### Exercise Training Protocol

Exercise training was performed on female syndecan-4^–/–^ mice (KO) and syndecan-4^+/+^ mice (WT) bred from the same genetic background with a treadmill for rodents (Columbus Instruments, OH, United States) with a 30-degree inclination. The mice were adapted 2 days to the treadmill, before exercise training was performed for 60 min per day for 14 days (1 day rest without training) on a treadmill with moderate intensity. All mice had 3 min of warmup before each running session that consisted of six times 8 min of running followed by 2 min rest. Running speed was set to 14–18 m per minute. Mice that were not able to complete the training protocol were excluded from the study. Animals were sacrificed by cardiac excision in deep surgical anesthesia.

### Real-Time PCR

Tibialis anterior muscle was collected from WT and syndecan-4^–/–^ mice, exercised and sedentary mice, and snap-frozen in liquid nitrogen. Tissue samples were homogenized in lysis RLT-buffer of RNeasy minikit (#74104, Qiagen, Hilden, Germany) using a Precellys24 (#74106, Bertin Technologies, Villeurbanne, France) at 5500 rpm for 2 × 20 s, and RNA further purified following the manufacturer’s protocol of the RNeasy minikit including a DNase treatment on column according to the manufacturer’s protocol. cDNA was generated from ∼400 ng mRNA using TaqMan^®^ Reverse Transcription Reagents (Invitrogen, Carlsbad, CA, United States) according to the manufacturer’s protocol. The cDNA was diluted four times before aliquots (in duplicates) were subjected to real-time PCR analysis using an ABI Prism 7700 Sequence Detection system (Applied Biosystem, United Kingdom), and TaqMan^®^ primer/probe assays (see Table 1 for primer/probes used) according to manufacturer’s protocol. The efficiency of each set of primers was always higher than 96%. Amplification of cDNA by 40 two-step cycles (15 s at 95°C for denaturation of DNA, 1 min at 60°C for primer annealing and extension) was used, and cycle threshold (Ct) values were obtained graphically (Applied Biosystem, Sequence Detection System, Software version 2.2). ΔCt values and ΔΔCt values were calculated according to the MIQE guidelines (Bustin et al., 2010). Comparison of the relative gene expression (fold change) was derived by using the comparative Ct method. In short, values were generated by subtracting ΔCt values between two samples which gives a ΔΔCt value. The relative gene expression (fold change) was then calculated by the formula 2^–ΔΔCt^ (Schmittgen and Livak, 2008). Statistical analyses were performed using the ΔΔCt-values.

**TABLE 1:** Gene target and TaqMan^®^ primer/probe assays.

Gene target	TaqMan^®^ primer/probe assays
Decorin	Mm00514535_m1
Fibromodulin	Mm00491215_m1
Lumican	Mm00500510_m1
Biglycan	Mm00455918_m1
Collagen 1a2	Mm00483888_m1
Collagen 3a1	Mm00802331_m1
Lox	Mm00495386_m1
Syndecan-2	Mm00484718_m1
Syndecan-4	Mm00488527_m1
Syndecan-3	Mm01179833_m1
MyoD	Mm01203489_g1
Desmin	Mm00802455_m1
Myogenin	Mm00446195_g1
Ribosomal protein gene RP132	Mm02528467_g1

### Immunofluorescence

TA muscles (*n* = 4) were collected from WT and syndecan-4^–/–^ mice, embedded in OCT compound (Tissue Tek, Sakura Finetek, CA, United States) and then snap-frozen. Five micrometer-thick sections were cut on a cryostat and mounted on poly-L-lysine coated glass slides. The sections were air-dried for 5 min, before fixation using ice-cold acetone for 5 min. The sections were washed briefly twice in PBS, permeabilized using 0.5% Triton-X in PBS and incubated with 5% non-fat dry milk for 30 min before incubation with primary antibody for 1 h. Subsequent incubation with secondary antibodies was performed for 30 min before mounting using Dako fluorescent mounting medium (Glostrup, Denmark). Antibodies used were: Rabbit anti-Collagen 1 (ab34710, Abcam, Cambridge, United Kingdom), anti-pSer473-Akt (#9271, Cell Signaling, United States) and Alexa 488-goat anti-rabbit (#A-11078 Thermo Fisher Scientific, MA, United States). To quantify myofiber number sections were air-dried for 30 min and incubated with P-Block (5% goat serum, 1% Triton-X, 0.012% BSA, 0.012% non-fat dry-milk in PBS) for 1h. The sections were washed briefly four times with P-Block before incubation with primary antibody over night at 4°C. Subsequent washing was performed twice with PBS before incubation with secondary antibody in P-Block for 1 h. The sections were washed briefly three times with P-Block, twice with PBS for 5 min each, rinsed in water before mounted using Dako fluorescent mounting medium. The total number of fibers in WT and KO TA muscles were quantified based on Laminin staining, using the ImageJ Cell counter plugin. Antibodies used were: Rabbit anti-Laminin (PA5-16287, Thermo Fisher Scientific, MA, United States). DyLight 549 mouse anti-rabbit were from Jackson Immunoresearch Cambridgeshire, United Kingdom. The sections were examined by fluorescence microscopy analysis (apotome mode) (ZEISS Axio Observer Z1 microscope, Jena, Germany), and images were processed using Adobe Photoshop CS3. If needed, brightness and contrast were adjusted manually across the entire image. Myofiber cross-sectional area in WT and KO TA muscles were quantified based on collagen staining. A minimum of 1,000 myofibers and four sections were analyzed per biological replica. Hoechst (Hoechst 33342, 10 μg/ml) was from Thermo Fisher Scientific. For assessment of connective tissue, sections were incubated with WGA Alexa Fluor^TM^ 546 Conjugate (Thermo Fisher Scientific, MA, United States).

### Western Blotting

Protein extracts from TA muscle were prepared using ice-cold lysis buffer (20 mm Hepes, pH 7.5, 150 mm NaCl, 1 mm EDTA, 0.5% Triton) supplemented with Complete EDTA-free protease inhibitor cocktail (#5056489001, Roche Applied Science, Merck, Darmstadt, Germany) and PhosSTOP (#4906837001, Roche Applied Science). Tissue samples were homogenized three times for 1 min on ice with a Polytron 1200 homogenizer and centrifuged at 14,000 × *g* for 10 min at 4°C. Supernatants were collected and stored at −70°C. Protein concentrations were determined using the Micro BCA protein assay kit (#23235, Pierce). An equal amount of protein was loaded per lane (40 μg/μl). The protein extracts were resolved on 4–15% Criterion^TM^ Tris-HCl precast gels (#3450029, Bio-Rad Laboratories, CA, United States) and blotted onto PVDF membranes (#1704157, Trans-Blot Turbo Transfer Pack, Bio-Rad) using the Trans-Blot Turbo system (Bio-Rad) or tank blotting. The PVDF membranes were blocked in 5% non-fat dry milk, 1% casein or 3% BSA in TBS-T for 60 min at room temperature, followed by incubation with primary antibodies overnight at 4°C. Membranes were washed two times for 10 min each in TBS-T, incubated with a horseradish-peroxidase-conjugated secondary antibody and thereafter washed two times for 10 min and one time for 5 min in TBS-T. Blots were developed using ECL Prime (RPN 2232, GE Healthcare, IL, United States). The chemiluminescence signals were detected by Las-4,000 (GE Healthcare). Membranes were re-probed after stripping using the Restore Western Blot Stripping buffer for 5 min at room temperature (21059, Thermo Fisher Scientific, MA, United States).

Antibodies and conditions were: anti-pSer235/236-RPS6 (1:1000, 5% BSA, #4858, Cell Signaling, MA, United States), anti-RPS6 (1:1000, 5% BSA, #2217, Cell Signaling), anti-HES-1 (1:500, 1% casein, #AB5702, Millipore, Merck, Darmstadt, Germany), anti-LRP6 (1:1000, 1% casein, #2560, Cell Signaling), anti-Dvl (1:1000, 1% casein, #3224, Cell Signaling), anti-β-catenin (1:5000, 1% casein, ab32572, Abcam, Cambridge, United Kingdom), anti-Frizzled-7 (1:1000, 1% casein, #ab64636, Abcam), anti-Cleaved Notch1 (1:1000, 5% BSA, #4147, Cell Signaling), anti-Wnt4 (1:1000, 1% casein, ab91226, Abcam), anti-TRPC7 (1:500, 1% casein, #SAB5200051, Sigma-Aldrich), anti-PKC (1:250, 1% casein, sc-208, Santa Cruz, TX, United States), anti-DSCR1/RCAN1.4 (1:500, 5% milk, #D6694, Sigma-Aldrich), anti-pSer240/244-RPS6 (1:1000, 5% BSA, #5364 Cell Signaling), anti-pSer473-Akt (1:500, 5% BSA, #9271, Cell Signaling), anti-pThr308-Akt (1:500, #5106, 5% BSA, Cell Signaling), anti-Akt (1:500, 1% casein, #9272, Cell Signaling), anti-fibromodulin (1:500, 1% casein, sc-33772, Santa Cruz), anti-pSer2448-mTOR (1:1000, #2971, 5% BSA, Cell Signaling), anti-mTOR (1:1000, #2983, 5% BSA, Cell Signaling), anti-Pax-7 (1:500, sc-81648, 1:500, 1x casein, Santa Cruz) anti-decorin (1:500, 1% casein, AF1060, R&D Systems, MN, United States), anti-biglycan (1:500, 1% casein, ab49701, Abcam) and anti-LOX (1:500, 1% casein, sc-32409, Santa Cruz). Anti-rabbit IgG HRP (NA934V) affinity-purified polyclonal antibody, anti-mouse IgG HRP (NA931V) (both from GE Healthcare) and anti-goat IgG HRP (HAF109, R&D Systems, MI, United States) were used as secondary antibodies. Since housekeeping genes like vinculin and beta-tubulin were regulated across the four groups, ProBlueSafeStain (Coomassie) (#G00PB001, Giotto Biotech, FI, Italy) was used to verify equal protein loading. Two or three distinct Coomassie stained bands, mostly in the 75–100 kDa region, was used to show equal loading of the samples.

### Statistics

All data were expressed as mean ± SEM. Comparisons between two groups were analyzed using Mann–Whitney *U* test (GraphPad Prism version 8.0.1, La Jolla, CA, United States). A *p*-value of <0.05 was considered statistically significant. Comparison of fiber area sizes was performed using analysis of variance where each fiber’s estimated area was used as a sample, WT/KO was used as a fixed effect (the effect of interest) and magnification and muscle image number were included as random effects. The reason for including magnification was that image generation and fiber identification could be affected by the magnification. Muscle image was included to separate individual mouse effects from the true fiber area effects. Analyses were conducted in MATLAB 2015a, The MathWorks, Inc., Natick, MA, United States.

## Results

### Syndecan-4^–/–^ Mice Have Reduced Body Weight, Smaller Muscle Fibers and Reduced myoD and Myogenin mRNA Levels

**FIGURE 1:**
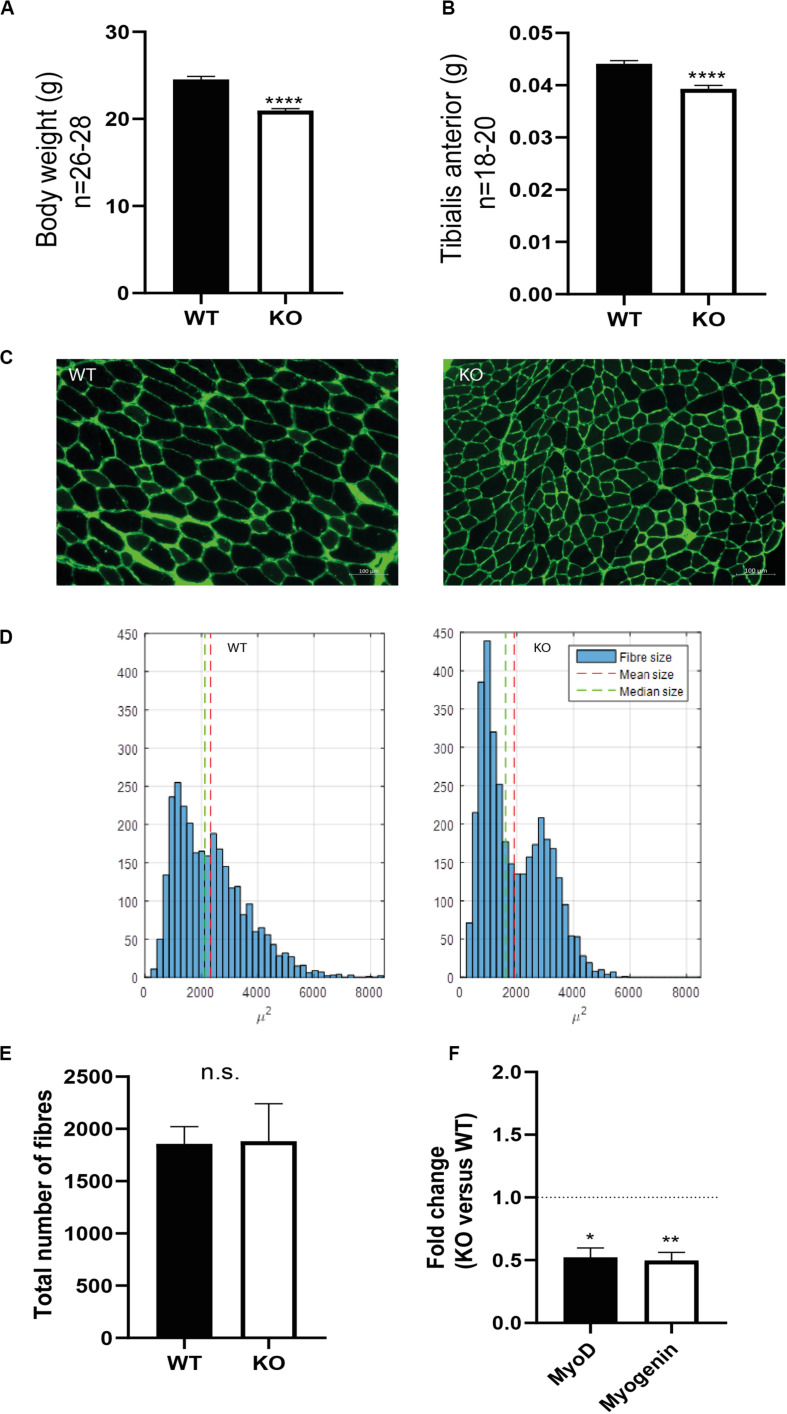
Syndecan-4^–/–^ mice have reduced body weight, smaller muscle fibers and reduced myoD and myogenin mRNA levels. Bars show **(A)** bodyweight and **(B)** tibialis anterior (TA) wet weight in syndecan-4^–/–^ mice compared to WT. The data are presented as average ± SEM. Asterisks denote significant differences (***p < 0.001) between syndecan-4^–/–^ and WT using un-paired two-tailed *t*-test. **(C)** Cross-sections of TA muscles from WT (left panel) and syndecan-4^–/–^ mice (right panel) were cryo-sectioned and stained with rabbit-anti collagen 1 (green), followed by Alexa Fluor 488-conjugated goat anti-rabbit before fluorescence microscopy analysis. **(D)** Quantification of images in **(C)** show fiber size distribution with a shift toward more fibers of low cross-sectional area (right panel) in syndecan-4^–/–^ compared to WT (left panel). Differences were tested using an analysis of variance with random effects. **(E)** Quantification of number of fibers in WT and syndecan-4^–/–^. The data are presented as average ± SEM. Differences were tested using un-paired two-tailed *t*-test (ns; non-significant). **(F)** Bars show relative gene expression levels (fold change) in syndecan-4^–/–^ versus WT (young mice, aged 12–22 weeks, *n* = 12) ± SEM. The values of the bars are presented as fold change (in ΔΔCT) of syndecan-4^–/–^ related to WT (the latter is set to 1 and is represented as dotted line). Asterisk indicate significant differences, statistics assessed for ΔΔCT values by unpaired two-tailed *t*-test (*p < 0.05, **p < 0.01).

To investigate the skeletal muscle of syndecan-4^–/–^ mice, we analyzed the TA muscles from adult female mice of 12–22 weeks. The syndecan-4^–/–^ mice were viable and showed no obvious postnatal abnormalities, however, they had a significantly lower body weight and TA weight compared to WT (Figures 1A,B, respectively). Closer inspection showed that the syndecan-4^–/–^ muscle fibers had smaller cross-sectional area compared with those of the WT mice (Figure 1C). More specifically, a larger fraction of the syndecan-4^–/–^ muscle fibers scored less than 2000 μm^2^ and there were hardly any larger muscle fibers (6000 μm^2^ and over) (Figure 1D). Statistical analyses showed that the average size of the syndecan-4^–/–^ muscle fibers was 468 μm^2^ smaller compared to WT (*p* = 0.0009), which was not accompanied by a change in total number of myofiber per muscle section (Figure 1E). As previously reported (Cornelison et al., 2004), the mRNA levels of the muscle transcriptional activator myoblast determination protein (myoD) and myogenin (this study) were significantly lower in syndecan-4^–/–^ compared to WT (Figure 1F). Consistent with the previous report (Cornelison et al., 2004), we did not observe any centrally nucleated myofibers in the syndecan-4^–/–^ (Supplementary Figure 1A), a characteristic often associated with muscle disorders (Cornelison et al., 2004; Folker and Baylies, 2013) and impaired maintenance of MuSC quiescence (Pisconti et al., 2016). We did neither observe any differences in the protein levels of the paired box transcription factor Pax7, which is a marker of quiescent MuSCs (Seale et al., 2000) (Supplementary Figure 1B). This is in line with Cornelison et al. (2004), who demonstrated no difference in the number of MuSCs in syndecan-4^–/–^ and WT.

### The Syndecan-4^–/–^ Muscle Has Several Changes in the ECM Components

**FIGURE 2:**
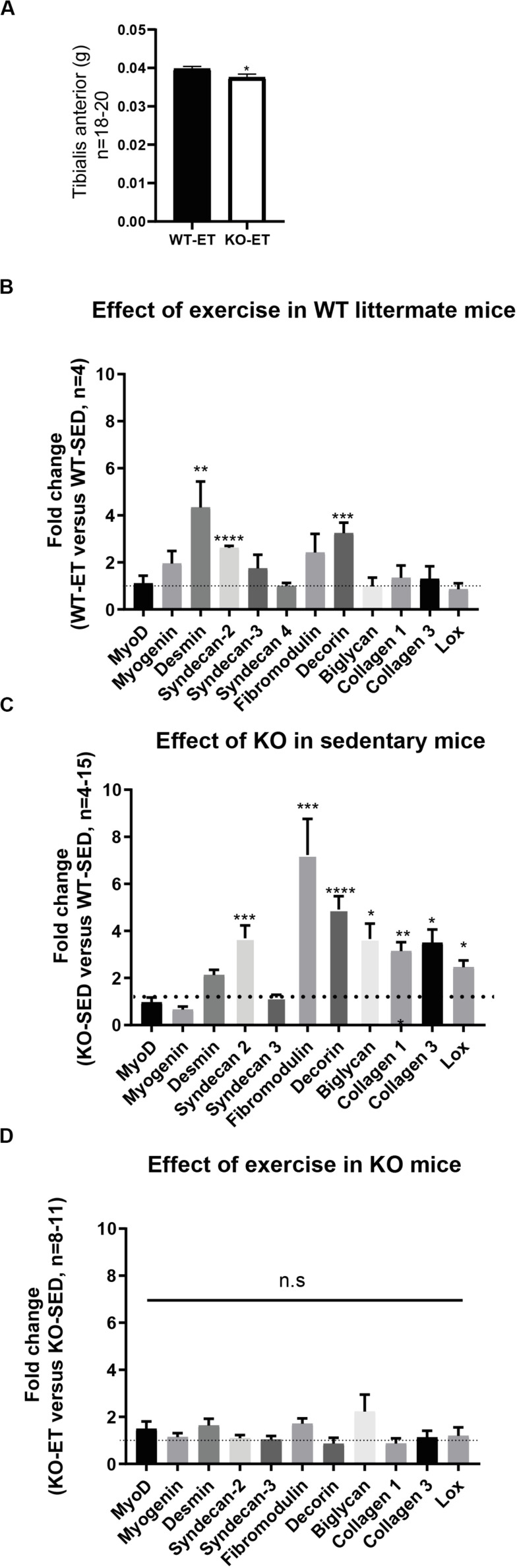
The syndecan-4^–/–^ muscle has increased mRNA levels of several ECM components. **(A)** Bars show tibialis anterior (TA) wet weight from exercised WT littermates (WT-ET) and syndecan-4^–/–^ mice (KO-ET) (10–12 weeks at harvest). The data is presented as the average ± SEM. Comparisons between the groups were analyzed using Mann–Whitney *U* test (^∗^*p* < 0.05). Bars show relative mRNA levels in TA muscle from **(B)** WT-ET versus WT-SED, **(C)** KO-SED versus WT-SED and **(D)** KO-ET versus KO-SED (10–12 weeks at harvest). The values of the bars are presented as fold change (ΔΔCT) in WT-ET/WT-SED, KO-SED/WT-SED and KO-ET/KO-SED in **(B–D)**, respectively. WT-SED is set to 1 in **(B,C)**, whereas KO-SED is set to 1 in **(D)** (represented as dotted lines). Statistics are based on ΔΔCT values using unpaired two-tailed *t*-test (*p < 0.05, **p < 0.01, ***p < 0.001).

To further investigate the role of syndecan-4, syndecan-4^–/–^ and age-matched WT littermates were subjected to a 2 weeks-long treadmill exercise protocol (ET mice) (10–12 weeks at harvest). Sedentary syndecan-4^–/–^ and WT littermates of same age were used as controls (SED mice). As shown in Figure 2A, the syndecan-4^–/–^ TA muscles were still significantly smaller than those from WT littermates after exercise (WT-ET versus KO-ET).

**FIGURE 3:**
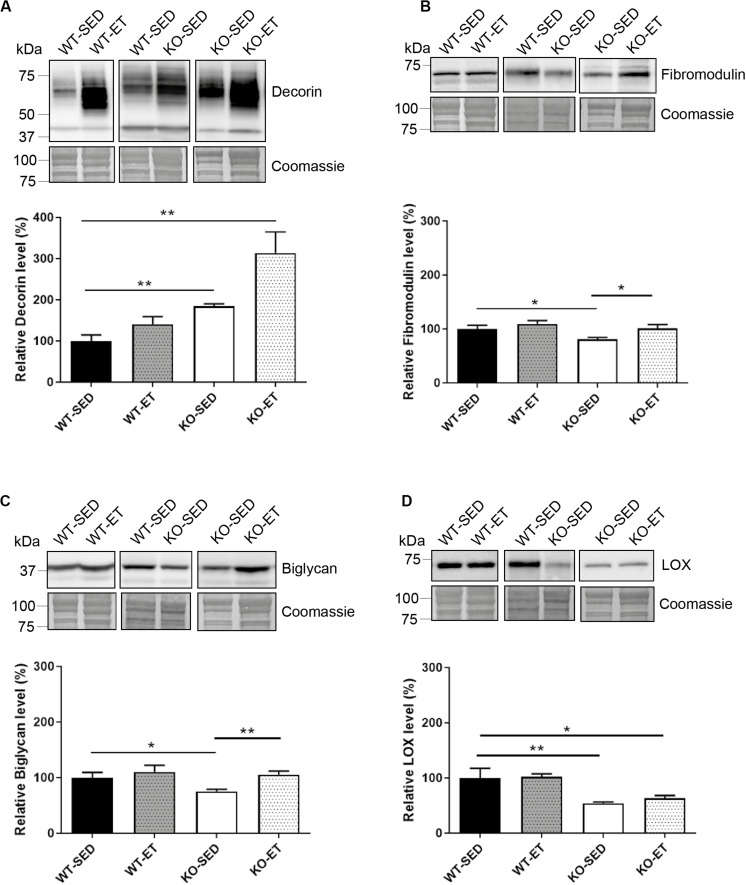
The syndecan-4^–/–^ muscle has changed protein levels of several ECM components. Immunoblot analyses of **(A)** decorin, **(B)** fibromodulin, **(C)** biglycan, and **(D)** LOX in WT-SED, WT-ET, KO-SED and KO-ET. The values are presented in percentage and are normalized to WT-SED (*n* = 4–10). Comparisons between the groups were analyzed using Mann–Whitney *U* test (*p < 0.05, **p < 0.01). ProBlue Safe Stain (Coomassie) was used as loading control.

To detect possible differences between syndecan-4^–/–^ and WT at the molecular level, we measured mRNA and protein levels of different ECM and signaling molecules we hypothesized could have a role in exercise and syndecan-4-mediated signaling. The mRNA levels of desmin and the ECM component decorin were both upregulated in WT after exercise (Figure 2B, WT-ET versus WT-SED), which is consistent with a role of decorin and desmin in myoblast differentiation and fusion (Brandan and Gutierrez, 2013; Hnia et al., 2015). Syndecan-2 was also highly up-regulated in response to exercise, while no changes were seen in syndecan-3 and 4 (Figure 2B). Myogenin and fibromodulin showed a tendency to increase in WT after exercise, whereas myoD, biglycan, collagen 1, collagen 3 and the collagen cross-linking enzyme lysyl oxidase (LOX) were unchanged (Figure 2B). Consistently, although not significant, immunoblotting indicated a slight increased level of decorin and unchanged levels of fibromodulin, biglycan and LOX after exercise (Figures 3A–D, WT-SED versus WT-ET).

Similar to exercise, syndecan-4^–/–^ loss led to increased mRNA levels of both syndecan-2 and decorin (Figure 2C, KO-SED versus WT-SED). However, in contrast to WT, exercise failed to induce any further upregulation (Figure 2D, KO-ET versus KO-SED). Interestingly, syndecan-4 loss also led to increased mRNA levels of ECM components and modifiers such as fibromodulin, biglycan, collagen 1, collagen 3 and LOX (Figure 2C, KO-SED versus WT-SED). Consistently with the mRNA data, immunoblotting showed increased levels of decorin when syndcan-4 was absent (Figure 3A, KO-SED versus WT-SED), but surprisingly a reduction in the fibromodulin, biglycan and LOX protein levels (Figures 3B–D, KO-SED versus WT-SED). Notably, the biglycan and LOX protein levels increased in syndecan-4^–/–^ mice in response to exercise (Figures 3C,D, KO-ET versus KO-SED), where fibromodulin and biglycan returned to the level observed in WT sedentary mice (Figures 3B,C, KO-ET versus WT-SED).

Taken together, the syndecan-4^–/–^ muscle had increased mRNA levels of syndecan-2, collagen 1 and 3, decorin, fibromodulin, biglycan, and LOX. These three latter ECM components were reduced at protein level, suggesting that they might be more susceptible to degradation or less efficiently translated when syndecan-4 is absent. Although exercise induced upregulation of desmin, syndecan-2 and decorin in WT mice, a further upregulation was not observed in exercised syndecan-4^–/–^ mice.

### The Syndecan-4^–/–^ Muscle Has Increased Akt/mTOR/S6K1 and Notch-HES-1 Pathways

**FIGURE 4:**
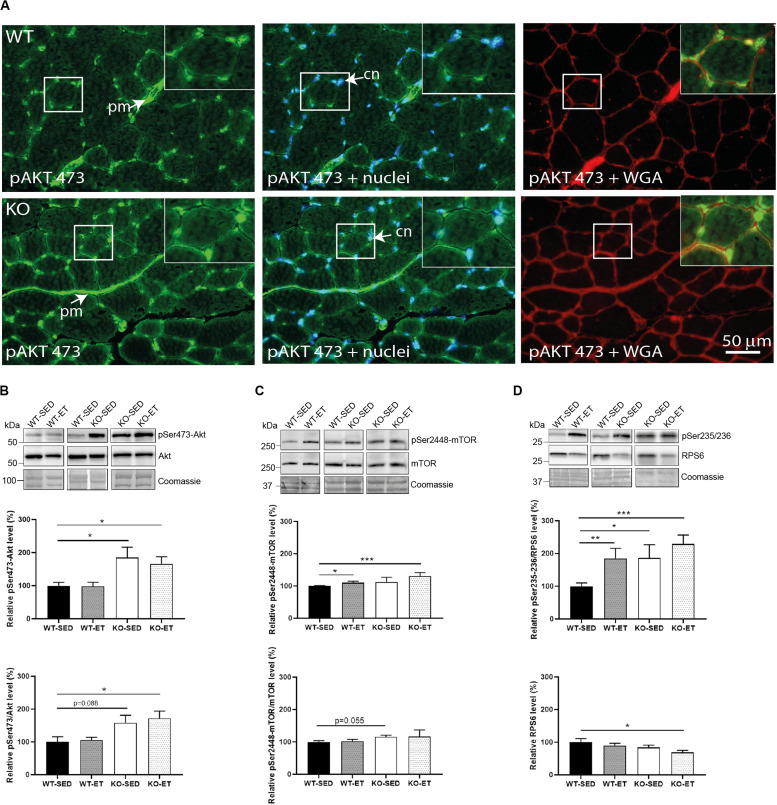
The syndecan-4^–/–^ muscle has an increased Akt/mTOR/S6K1 pathway. **(A)** Immunofluorescence of pSer473-Akt (green) in WT and syndecan-4^–/–^ muscles. The cell nuclei were counterstained with Hoechst (blue). The connective tissue was stained with WGA (red). *cn* cell nuclei, *pm* perimysium, indicated with arrows. Inserts show magnification of boxed areas. Scalebar as indicated. Immunoblot analyses of **(B)** pSer473-Akt (upper panels) and Akt (middle panels), **(C)** pSer2448-mTOR (upper panels) and mTOR (middle panels), and **(D)** pSer235/236-RPS6 (upper panels) and RPS6 (middle panels) in WT-SED, WT-ET, KO-SED and KO-ET. The values are presented in percentage and are normalized to WT-SED (*n* = 6–10). Comparisons between the groups were analyzed using Mann–Whitney *U* test (*p < 0.05, **p < 0.01 and ***p < 0.001). ProBlue Safe Stain (Coomassie) was used as loading control (lower panels in **B–D**).

Next, we analyzed activation of the protein kinase Akt, which transduces decorin-insulin-like growth factor I receptor (IGF1R) signaling (Suzuki et al., 2013) and regulates muscle fiber growth and hypertrophy through the Akt/mTOR/S6K1 pathway (Schiaffino et al., 2013). Interestingly, the Akt/mTOR pathway associates also with syndecan-4 signaling (Elfenbein and Simons, 2013). Immunofluorescence data showed that pSer473-Akt localized to the skeletal muscle cell nuclei and to the ECM perimysium in WT where fibroblasts and resident immune cells are located (Figure 4A, upper panels). Visual inspection clearly showed an increased expression of pSer473 in syndecan-4^–/–^ (Figure 4A, lower panels). Consistently, immunoblotting revealed that the pSer473-Akt levels were constitutively higher in both sedentary as well as exercised syndecan-4^–/–^ mice compared to WT mice (Figure 4B, KO-SED and KO-ET versus WT-SED). Also the levels of pThr308-Akt/Akt and pSer2448-mTOR/mTOR showed a tendency to be increased in sedentary syndecan-4^–/–^ mice (Supplementary Figure 2A and Figure 4C, KO-SED versus WT-SED). We also analyzed the phosphorylation levels of the 40S ribosomal protein S6 (RPS6), which is downstream of the Akt/mTOR/S6K1 signaling, and directly links to cell size control and myofiber growth (Ruvinsky et al., 2009; Meyuhas, 2015). Immunoblotting revealed that the pSer235/236-RPS6 levels were increased in WT muscles upon exercise (Figure 4D, WT-ET versus WT-SED), but also in sedentary and exercised syndecan-4^–/–^ (Figure 4D, KO-SED and KO-ET versus WT-SED). The levels of pSer240/244-RPS6 were also slightly increased in syndecan-4^–/–^ muscles, although the increase was not statistically significant (Supplementary Figure 2B, KO-SED versus WT-SED). No difference in the total RPS6 levels was observed between syndecan-4^–/–^ and WT muscles (Figure 4D, WT-SED versus KO-SED, lower most panel).

**FIGURE 5:**
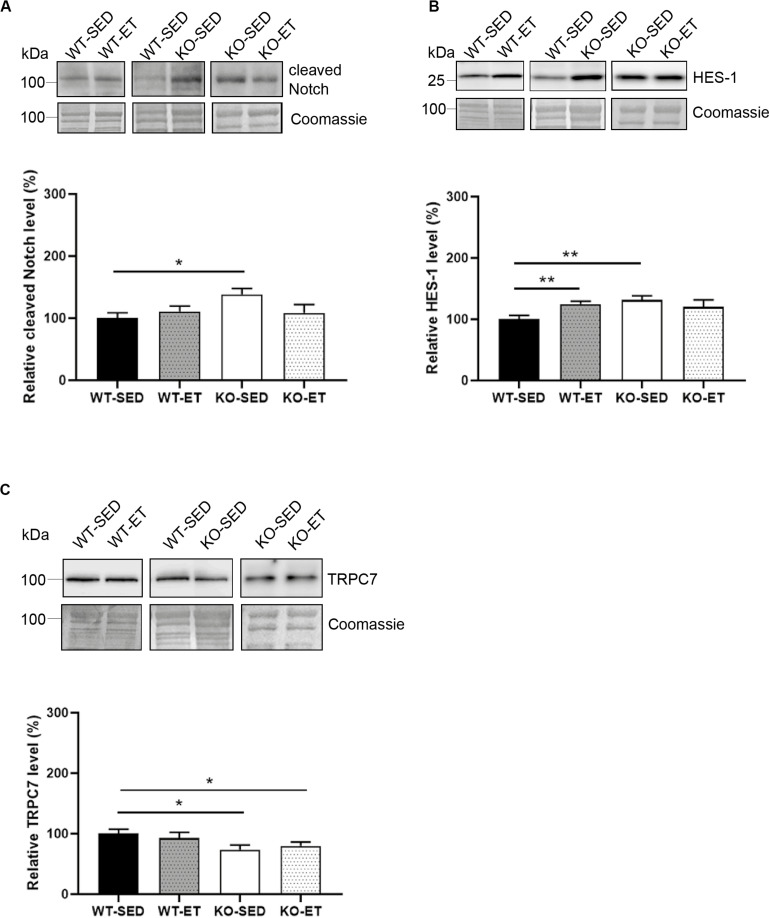
The syndecan-4^–/–^ muscle has an increased Notch/HES-1 pathway and a reduced TRPC7 level. Immunoblot analyses of **(A)** Cleaved Notch, **(B)** HES-1, and **(C)** TRPC7 in WT-SED, WT-ET, KO-SED, and KO-ET. The values are presented in percentage and are normalized to WT-SED (*n* = 6–10). Comparisons between the groups were analyzed using Mann–Whitney *U* test (*p < 0.05, **p < 0.01). ProBlue Safe Stain (Coomassie) was used as loading control (lower panels in **A–C**).

We next analyzed Cleaved Notch, as Notch signaling associates with increased syndecan-2 levels (Zhao et al., 2012), and is known to increase upon exercise and be involved in myogenesis (Arthur and Cooley, 2012). Although our data indicated no significant changes of Cleaved Notch1 in WT (Figure 5A, WT-ET versus WT-SED), the Notch target gene HES-1 was increased in response to exercise (Figure 5B, WT-ET versus WT-SED). Surprisingly, both the Cleaved Notch and HES-1 levels were increased in sedentary syndecan-4^–/–^ mice and did not further increase in response to exercise (Figures 5A,B, KO-SED versus WT-SED).

Finally, since syndecan-4 has been shown to associate with Wnt (Pataki et al., 2015), calcineurin-NFAT (Finsen et al., 2011) and protein kinase C (PKC) – transient receptor potential canonical 7 (TRPC7) signaling (Gopal et al., 2010), we also analyzed proteins in these pathways. Immunoblotting showed that the TRPC7 protein level was reduced in both sedentary and exercised syndecan-4^–/–^ (Figure 5C, KO-SED and KO-ET versus WT-SED). However, no differences were detected in disheveled (Dvl), β-catenin, frizzled-7, low-density lipoprotein receptor-related protein 6 (LRP6) or Wnt4 in syndecan-4^–/–^ versus WT, indicating no involvement of canonical Wnt/β-catenin or non-canonical Wnt signaling (Supplementary Figures 2C–G). Similarly, no differences in the PKC or RCAN4.1 (regulator of calcineurin and a target for NFAT) levels were detected (Supplementary Figures 2H,I).

Taken together, our data indicate that the syndecan-4^–/–^ muscle had increased activation of the Akt/mTOR/S6K1 and Notch-HES-1 pathways and that these pathways were not induced any further by exercise. Except for an also reduced TRPC7 level, few other changes were observed.

## Discussion

In this study we have characterized the TA muscle from syndecan-4^–/–^ mice and analyzed molecules involved in ECM remodeling and muscle growth, both under steady state conditions and in response to exercise. Compared to WT, we found that syndecan-4^–/–^ mice had reduced body weight, reduced muscle weight, muscle fibers with a smaller cross-sectional area, and reduced expression of MRFs. The syndecan-4^–/–^ mice had also increased activation of the Akt/mTOR/S6K1 and Notch-HES-1 pathways, a reduced TRPC7 level, increased syndecan-2 expression, and altered expression of several ECM components. These molecular changes were observed under steady state conditions, and in contrast to WT mice, were mostly not affected further by exercise.

**FIGURE 6:**
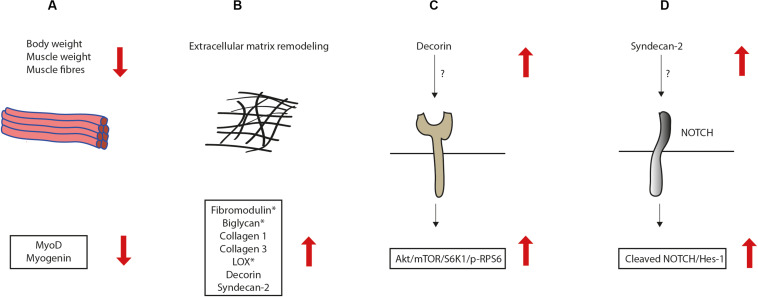
Illustration of alterations in the syndecan-4^–/–^ mice. **(A)** The syndecan-4^–/–^ mice had reduced body weight, muscle weight, muscle fibers and expression of myogenic regulatory transcription factors. **(B)** The syndecan-4^–/–^ muscle had increased mRNA levels of syndecan-2 and the ECM components decorin, fibromodulin, biglycan, collagen 1, collagen 3 and the collagen cross-linking enzyme lysyl oxidase (LOX). However, fibromodulin, biglycan and LOX were reduced at the protein level (denoted with star), suggesting that these ECM components are more susceptible to degradation or less efficiently translated when syndecan-4 is absent. The syndecan-4^–/–^ muscle had also increased activation of the **(C)** Akt/mTOR/S6K1 and **(D)** Cleaved Notch1-HES-1 pathways.

Closer inspection of the syndecan-4^–/–^ muscle showed that the muscle fibers were on average smaller than wild type (WT) fibers, which probably accounts for the reduced TA weight. Consistent with previous findings (Cornelison et al., 2004), we found that myoD and myogenin were reduced in syndecan-4^–/–^ mice of 12–22 weeks. MyoD is required for MuSC activation, proliferation and differentiation (Asfour et al., 2018). Myogenin expression is upregulated during differentiation and directs differentiating myoblasts to become terminally differentiated (Asfour et al., 2018). Consistent with a role of syndecan-4 in myogenesis, we have previously also demonstrated that the cytoplasmic part of syndecan-4 plays an important role in the early fusion process of myoblasts *in vitro* (Rønning et al., 2015). Our findings of the reduced body weight, muscle weight, muscle fibers and MRFs in syndecan-4^–/–^ are illustrated in Figure 6A.

Previous experiments have shown that both syndecan-3 and syndecan-4 are affected by exercise in humans. Syndecan-4 was reported up-regulated after acute exercise, whereas syndecan-3 was up-regulated after long-term exercise (Hjorth et al., 2015). However, in another study using rats, exercise did not change the syndecan-4 level (Eftestol et al., 2016). In our study using mice subjected to 2 weeks treadmill running, syndecan-2, but not syndecan-4 was increased, suggesting a potential involvement of syndecan-2 in the muscle response to exercise. Interestingly, we also observed higher levels of syndecan-2 in syndecan-4^–/–^ compared to WT (illustrated in Figure 6B). Upregulation of syndecan-2 has been reported as a compensatory mechanism during cartilage development in syndecan-4^–/–^ (Bertrand et al., 2013). However, syndecan-2 was not higher in the left ventricle from syndecan-4^–/–^ compared to WT (Strand et al., 2013), suggesting that compensatory upregulation of syndecan-2 is tissue dependent.

Although the mRNA levels of fibromodulin, decorin, biglycan, collagen 1, collagen 3 and the collagen cross-linking enzyme LOX were increased in syndecan-4^–/–^ mice, the protein levels of fibromodulin, biglycan and LOX were reduced (illustrated in Figure 6B). This finding might suggest that some ECM components are more susceptible to degradation or less efficiently translated when syndecan-4 is absent. Indeed, proteins involved in the translational machinery have been identified in the cardiac syndecan-4 interactome (Mathiesen et al., 2019). It is also possible that syndecan-4 affects factors involved in post-transcriptional regulation of these proteins. Decorin is abundant in adult skeletal muscle and expressed during skeletal muscle differentiation (Brandan and Gutierrez, 2013), and consistent with our mice mRNA data, reported to be upregulated in rats after moderate treadmill running (Xu et al., 2018). Biglycan expression is reported highly upregulated during injury-induced regeneration, both in growing myotubes and in activated precursor cells, then the expression decreases again during maturation of muscle fibers (Casar et al., 2004; Brandan and Gutierrez, 2013). The relationship between biglycan and syndecan-4 in skeletal muscle is not known, however a recent publication using cultured vascular endothelial cells suggests biglycan as a regulatory molecule of the ALK5-Smad2/3-TGF-β1 signaling, with a direct negative impact on syndecan-4 expression (Takato et al., 2017). Further experiments are required to investigate whether the ALK5-Smad2/3-TGF-β1 pathway is also involved in the biglycan-syndecan-4 relationship in skeletal muscle. Fibromodulin is also central in the myogenic program and plays an important role in tissue repair (Lee et al., 2016). Specifically, fibromodulin triggers myoblast differentiation by preventing myostatin binding to its receptor; the activin receptor type-2B. Fibromodulin, biglycan and decorin are also suggested to regulate collagen fibrillogenesis and crosslinking, and thus ECM integrity and function of skeletal muscle (Kalamajski and Oldberg, 2010). Whether the increased mRNA expression of these ECM components, including collagens and LOX, represent a compensatory mechanism to improve ECM function and trigger myoblast differentiation in the syndecan-4^–/–^ muscle, has to be determined in future work. In contrast to our findings, the syndecan-4^–/–^ heart has rather reduced mRNA levels of collagen 1, collagen 3 and LOX following pressure overload, leading to an impaired cross-linking and collagen assembly (Herum et al., 2013, 2015). Our group has also previously shown that syndecan-4 is essential for cardiac myoblast differentiation (Herum et al., 2013) and that the syndecan-4^–/–^ hearts do not develop concentric hypertrophy, but rather left ventricle dilatation and dysfunction following pressure overload (Finsen et al., 2011). Although a syndecan-4-calcineurin-NFAT pathway appeared essential for these events in the heart (Finsen et al., 2011; Herum et al., 2013, 2015), we found no changes in the calcineurin-NFAT pathway in the syndecan-4^–/–^ TA muscle. Thus, although syndecan-4 is involved in both differentiation and cell growth of the heart and skeletal muscle, different pathways seem to be involved.

We also found higher levels of pSer473-Akt and pSer235/236-RPS6 in syndecan-4^–/–^. The Akt/mTOR pathway is an important signaling pathway promoting muscle growth and fibrosis (Bodine et al., 2001; Li et al., 2013). Akt induces protein synthesis and hypertrophy by activating the serine/threonine kinase mTOR and the ribosomal protein S6 kinase 1 (SK61) (Schiaffino et al., 2013). SK61 is one of the main kinases phosphorylating ribosomal protein 6 (RPS6) (Meyuhas, 2015). Although RPS6 is primarily a structural protein of the ribosome, phosphorylation of RPS6 has been shown to be directly involved in cell size control (Meyuhas, 2015), and affects both translation as well as transcription efficiency. Our finding of an increased pSer235/236-RPS6 level in the syndecan-4^–/–^ mice was quite surprising, since the smaller cell size and smaller cross-sectional area of myofibers rather links to a reduced pSer235/236-RPS6 level (Ruvinsky et al., 2009; Meyuhas, 2015). Our finding of the increased activation of the Akt/mTOR/S6K1 pathway in syndecan-4^–/–^ is illustrated in Figure 6C. Notably, decorin, which was also increased in syndecan-4^–/–^, has been shown to activate Akt downstream of IGF1R, in addition to increase myoD and myogenin expression and promote myoblast differentiation (Suzuki et al., 2013).

Our findings of higher levels of both Cleaved Notch and HES-1 in syndecan-4^–/–^ mice, strongly suggest an increased Notch signaling when syndecan-4 is absent. HES-1 signaling was also higher in exercised compared to sedentary WT mice. Notch is a regulator of myogenesis and is increased upon physiological stimuli such as exercise (see review Arthur and Cooley, 2012; LaFoya et al., 2016). Notch signaling is indispensable for maintaining quiescence and self-renewal of MuSCs, and promotes proliferation of MuSCs by keeping low MyoD levels and prevents differentiation (Buas and Kadesch, 2010; Bi et al., 2016). Upon ligand binding, the intracellular domain of the Notch receptor is cleaved off (Cleaved Notch) and translocated to the nucleus where it activates the transcription factor CLS/RBPJ and induces expression of the transcriptional repressors of the HES and HEY family (Iso et al., 2003; D’Souza et al., 2008). Interestingly, both syndecan-2 and 3 have been linked to Notch signaling pathways (Pisconti et al., 2010; LaFoya et al., 2016). Syndecan-3 interacts with Notch at the plasma membrane in the MuSC, and this interaction has been suggested to be important for Notch cleavage (Pisconti et al., 2010). Loss of syndecan-3 impairs Notch signaling, alters MuSC homeostasis and leads to progressively increased myofiber size, which is especially noticed in repeatedly injured and dystrophic mice (Pisconti et al., 2010, 2016). Syndecan-2 is reported highly abundant in early-differentiated myoblasts (Do et al., 2015). Interestingly, Cleaved Notch has been shown to induce syndecan-2 expression in smooth muscle cells, which again interacts with the Notch receptor at the surface membrane and promotes further Notch signaling (Zhao et al., 2012). Whether the increased Notch signaling we observed in syndecan-4^–/–^ is due to the increased syndecan-2 level or *vice versa* has to be determined by future experiments (illustrated in Figure 6D).

Desmin was the only protein in our analyses that was increased in exercised WT, but neither changed in sedentary or exercised syndecan-4^–/–^. Desmin is the most abundant intermediate filament protein in adult skeletal muscle, expressed at low levels in MuSCs and increased during myogenic commitment and differentiation (Hnia et al., 2015). Human heterozygous desmin mutations link to muscular weakness and different skeletal and cardiac myopathies. In line with this, the desmin^–/–^ mouse model is also reported weaker; it fatigued more easily and showed an impaired performance on endurance tests (Haubold et al., 2003). Whether the syndecan-4^–/–^ mice also fatigues more easily must be determined in larger endurance tests in future.

Conclusively, our data show that key molecular pathways involved in matrix remodeling and muscle growth were affected in syndecan-4^–/–^ mice. These molecular changes were observed under steady state conditions, and in contrast to WT, were mostly not affected by exercise. Functional studies to elucidate the exact role of the ECM components in the syndecan-4^–/–^ mice should be performed in future studies. In particular, compensatory effects of syndecan-2 and vice-versa is of particular interest in follow up-studies.

## Data Availability Statement

All datasets presented in this study are included in the article/Supplementary Material.

## Ethics Statement

All animal experiments were performed in accordance with the National Regulation on Animal Experimentation in accordance with an approved protocol and the Norwegian Animal Welfare Act and conform the NIH guidelines.

## Author Contributions

SR, CC, and MP: conceptualization, investigation, methodology, visualization, and writing the original draft. JA: conceptualization, investigation, methodology, and review and editing the original draft. AP: conceptualization, visualization, review and editing the original draft. VH, ML, and KL: investigation, methodology, and review and editing the original draft. IS: methodology, review and editing the original draft, and funding acquisition. SK: conceptualization, investigation, review and editing the original draft. GC: conceptualization, investigation, review and editing the original draft, and funding acquisition. All authors contributed to the article and approved the submitted version.

## Conflict of Interest

The authors declare that the research was conducted in the absence of any commercial or financial relationships that could be construed as a potential conflict of interest.

## Abbreviations

Dvl: disheveled
ECM: extracellular matrix
ET: exercised trained
GAG: glycosaminoglycan
IGF1R: insulin-like growth factor I receptor
LOX: Lysyl oxidase
LRP6: low-density lipoprotein receptor-related protein 6
MRFs: myogenic regulatory transcription factors
MuSC: skeletal muscle stem cells
Myf: myogenic factor
MyoD: myoblast determination protein 1
PKC: protein kinase C
RPS6: ribosomal protein 6
SED: sedentary
SK61: ribosomal protein S6 kinase 1
SLRPs: small leucine-rich proteoglycans
TA: tibialis anterior
TRPC7: Transient Receptor Potential Cation Channel Subfamily C Member 7
WT: wild type.
