# Supplementary material for: Syndecan-4–/– Mice Have Smaller Muscle Fibers, Increased Akt/mTOR/S6K1 and Notch/HES-1 Pathways, and Alterations in Extracellular Matrix Components
Source: Front Cell Dev Biol. 2020 Jul 31;8:730. doi: 10.3389/fcell.2020.00730 (PMC7411008; doi:10.3389/fcell.2020.00730)
Supplement: Supplementary file 1 [file Data_Sheet_1.PDF]

Fig. 3A (Decorin)

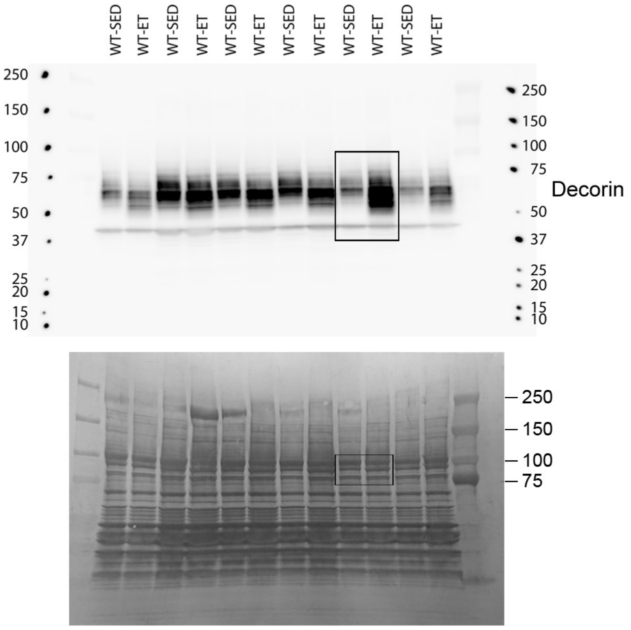

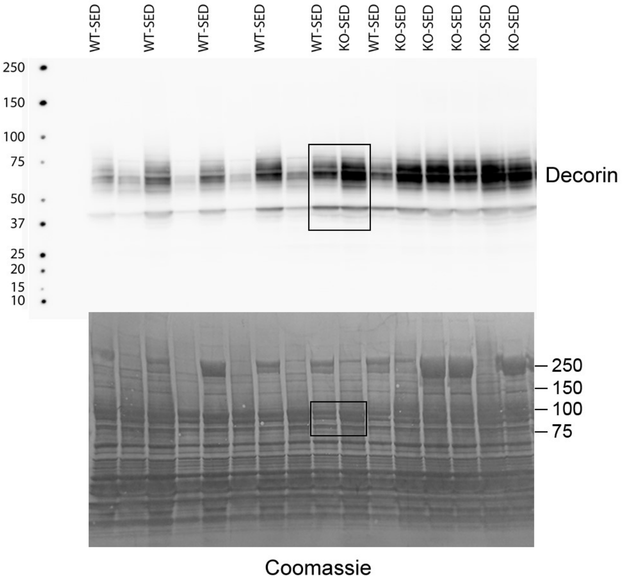

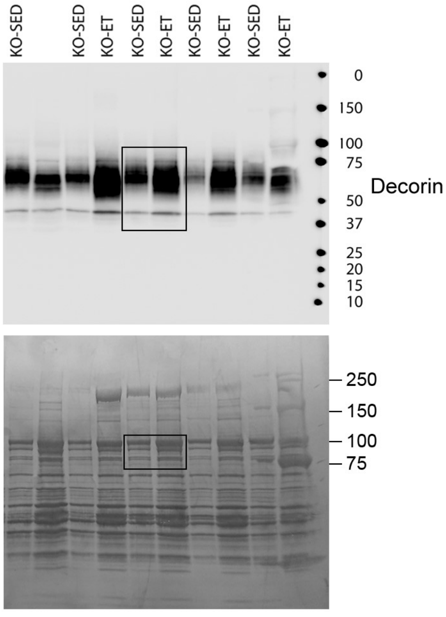

Since decorin seemed to be sensitive for the isolation procedure, only samples prepared at the same time were included in the analysis

Fig. 3B (Fibromodulin)

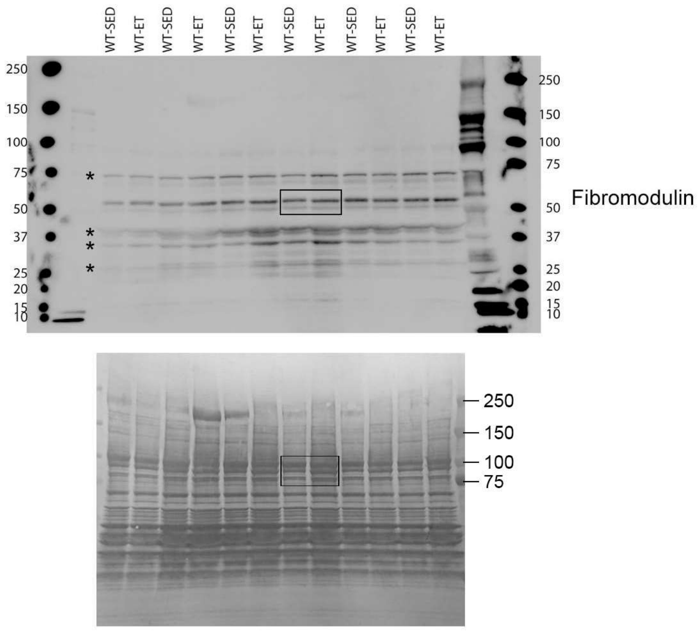

Coomassie

* Unspecific binding

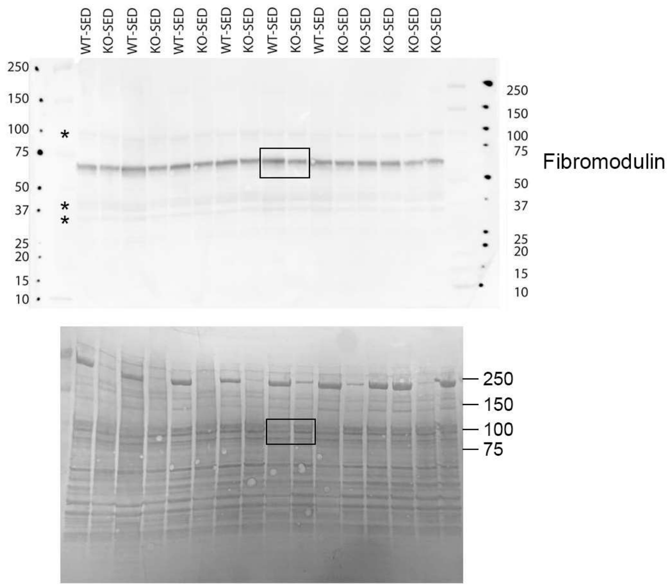

Coomassie

* Unspecific binding

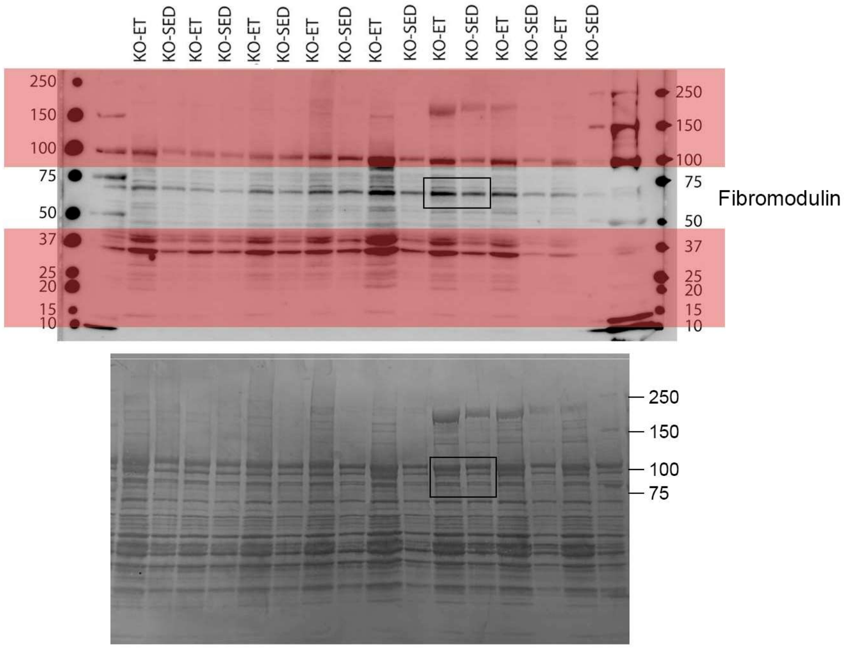

Coomassie

Strong bands in red boxes are background from another antibody.
KO-SED and KO-ET: The boxed samples are flipped horizontally in Fig. 3B.

Fig. 3C (Biglycan)

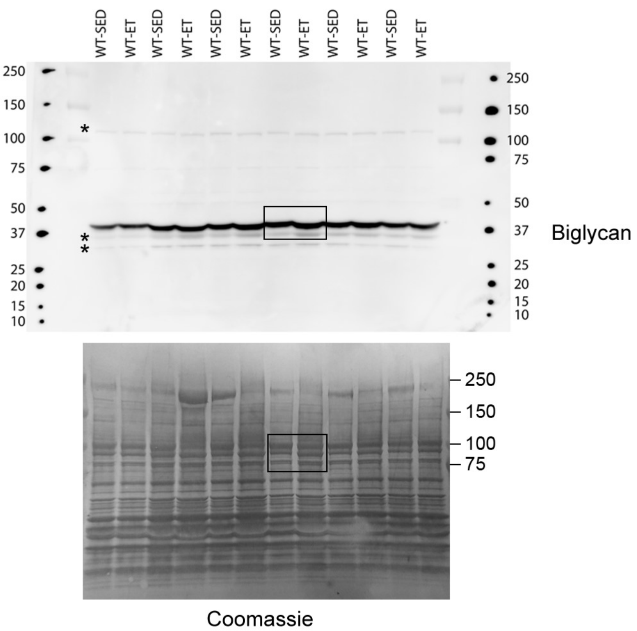

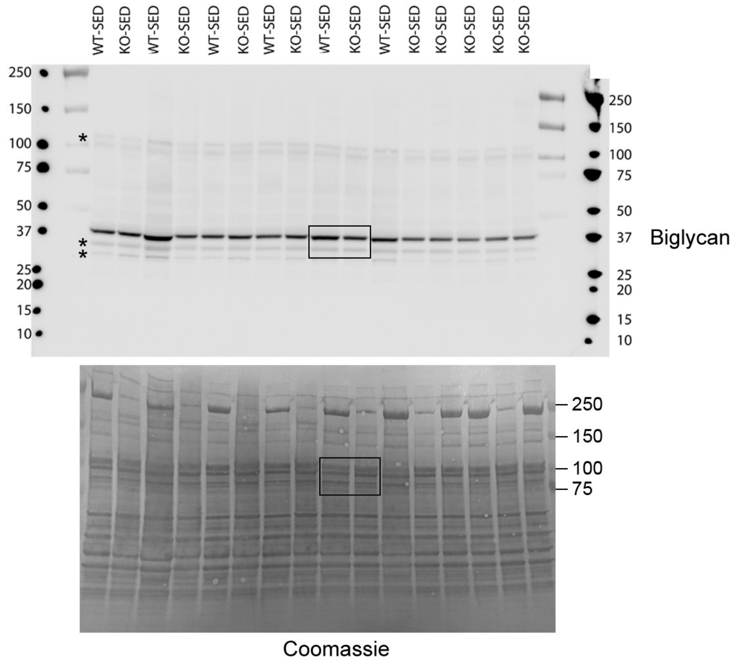

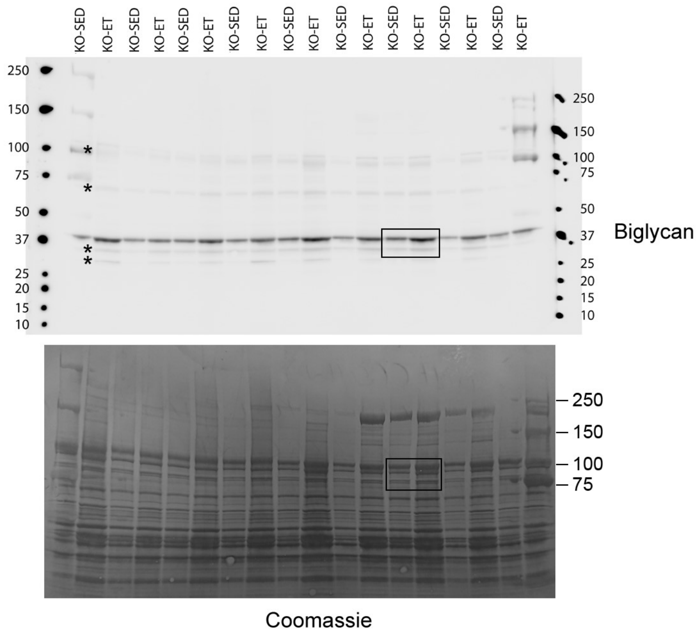

* Unspecific binding

Fig. 3D (LOX)

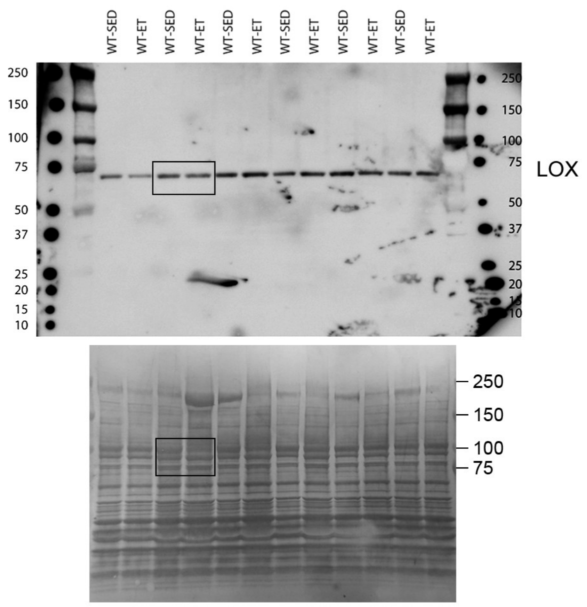

Coomassie

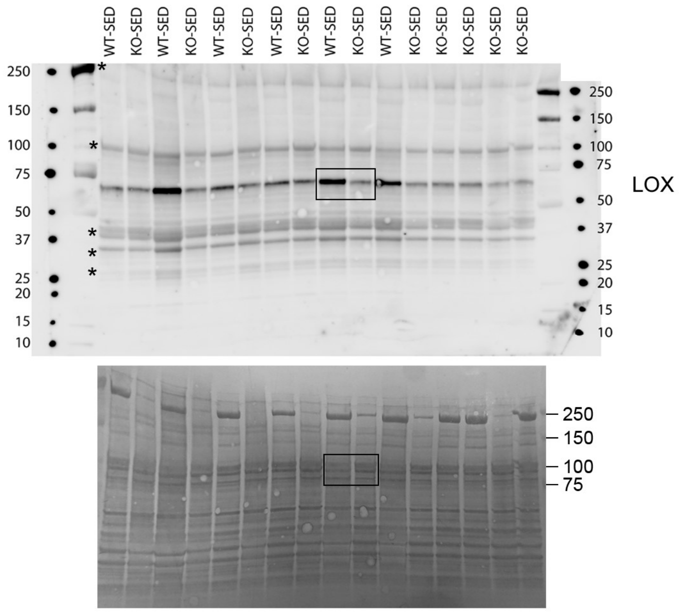

Coomassie

* Unspecific binding

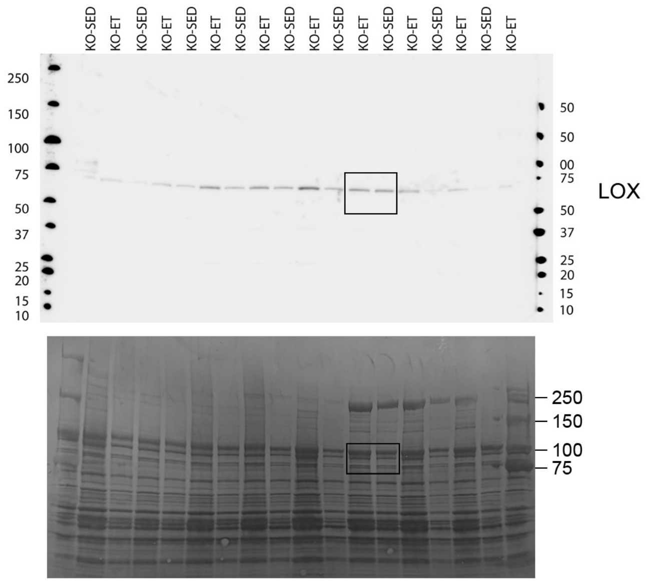

Coomassie

KO-SED vs KO-ET: The boxed samples are flipped horizontally in Fig. 3D.

Fig. 4B (pSer473-Akt)

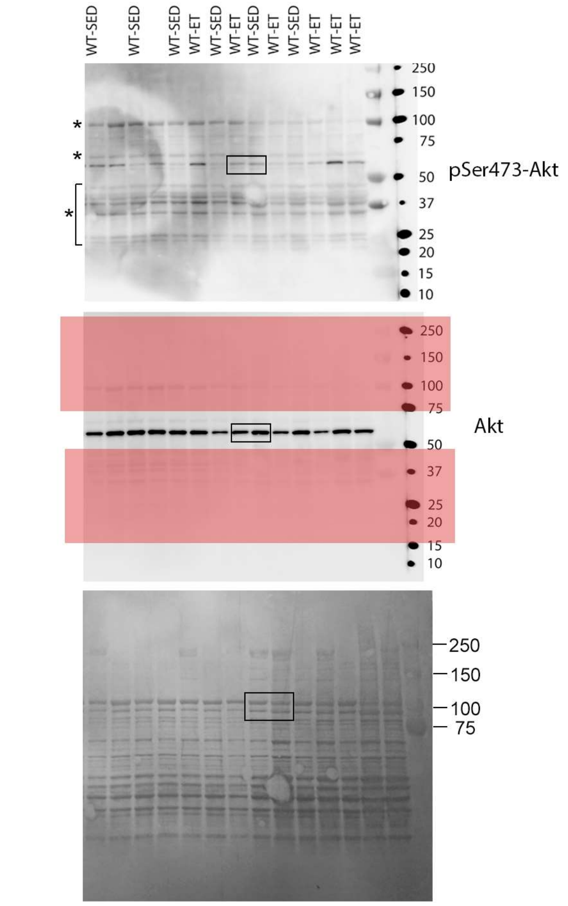

Coomassie

WT-SED and WT-ET: The boxed samples are flipped horizontally in Fig. 4A. Non-littermates are removed.

Bands in red boxes are background from anti-pSer473

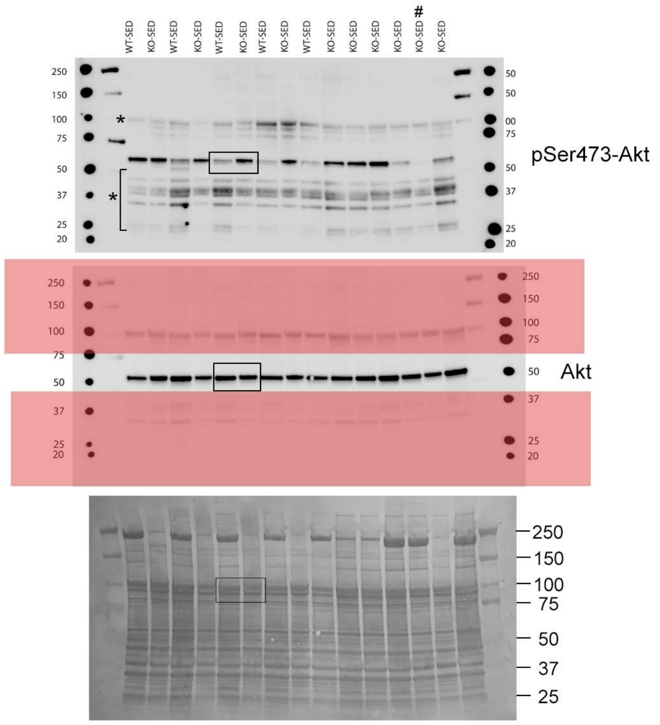

Coomassie

Sample excluded due to complete dephosphorylation

Bands in red boxes are background from anti-pSer473

* Unspecific binding

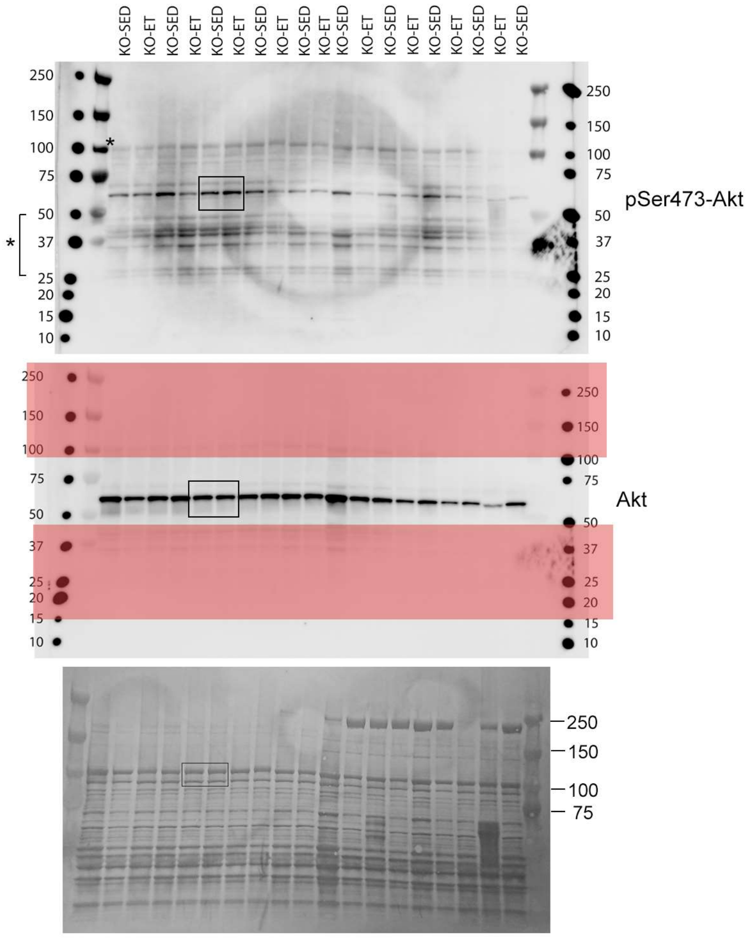

Coomassie

Bands in red boxes are background from anti-pSer473

Fig. 4C (pSer2448-mTOR)

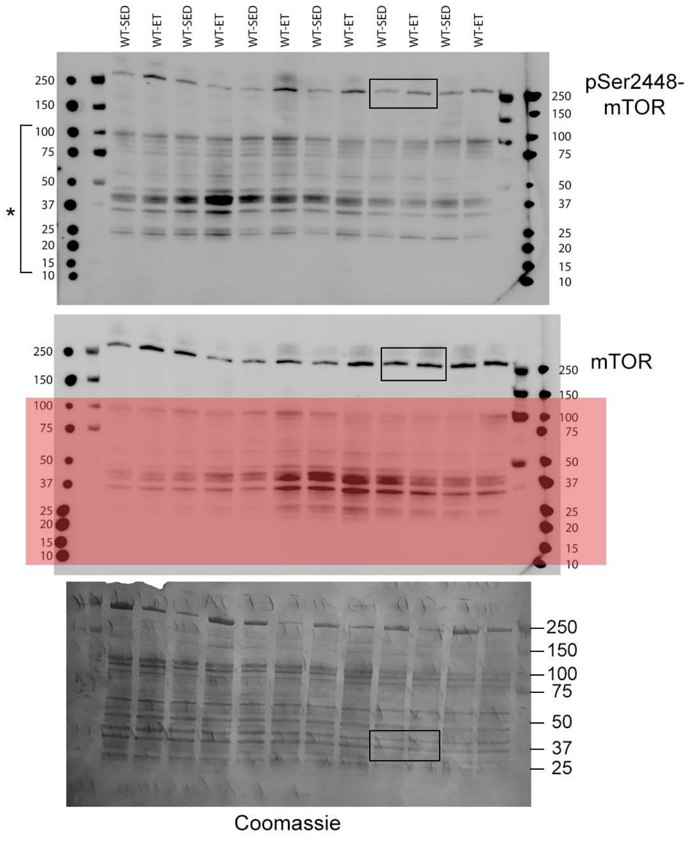

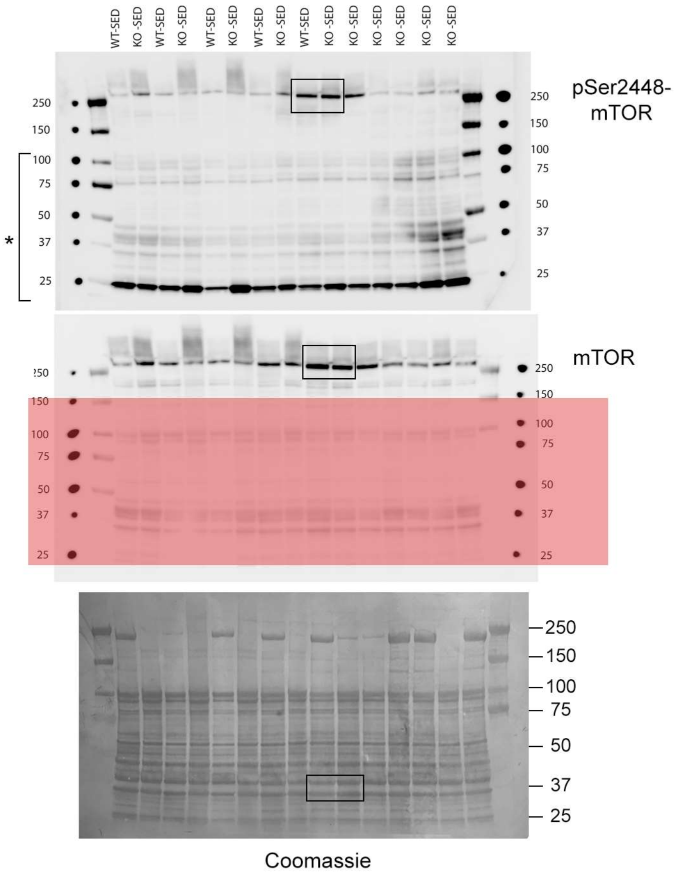

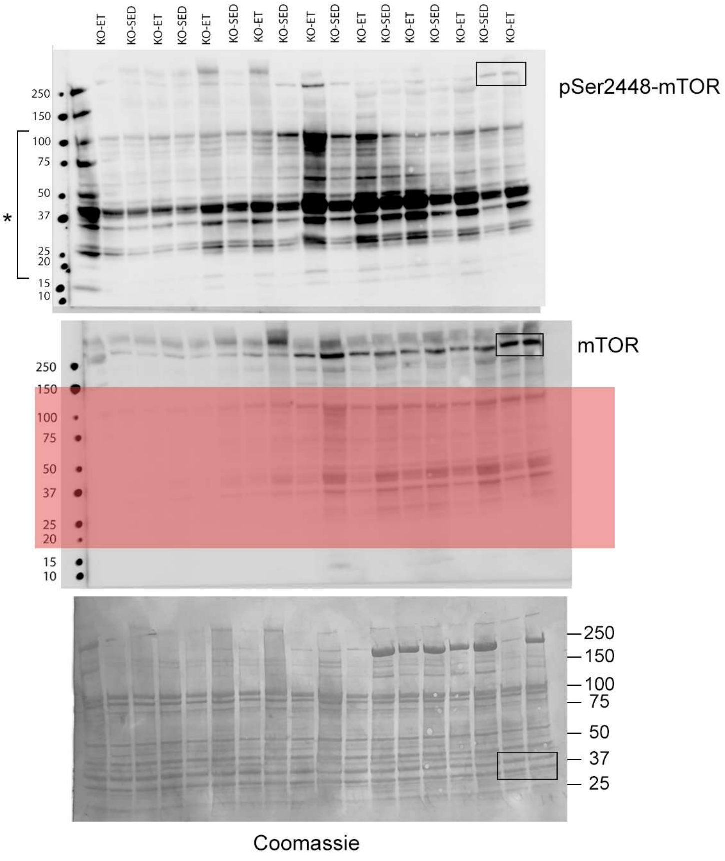

* Unspecific binding

Bands in red boxes are background from pSer2448-mTOR

Fig. 4D (pSer235/236-RPS6)

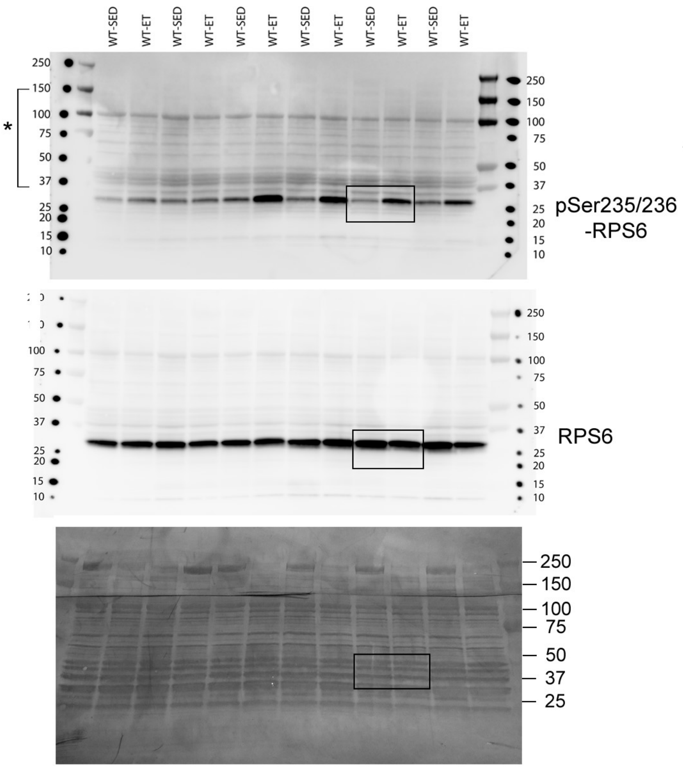

Coomassie

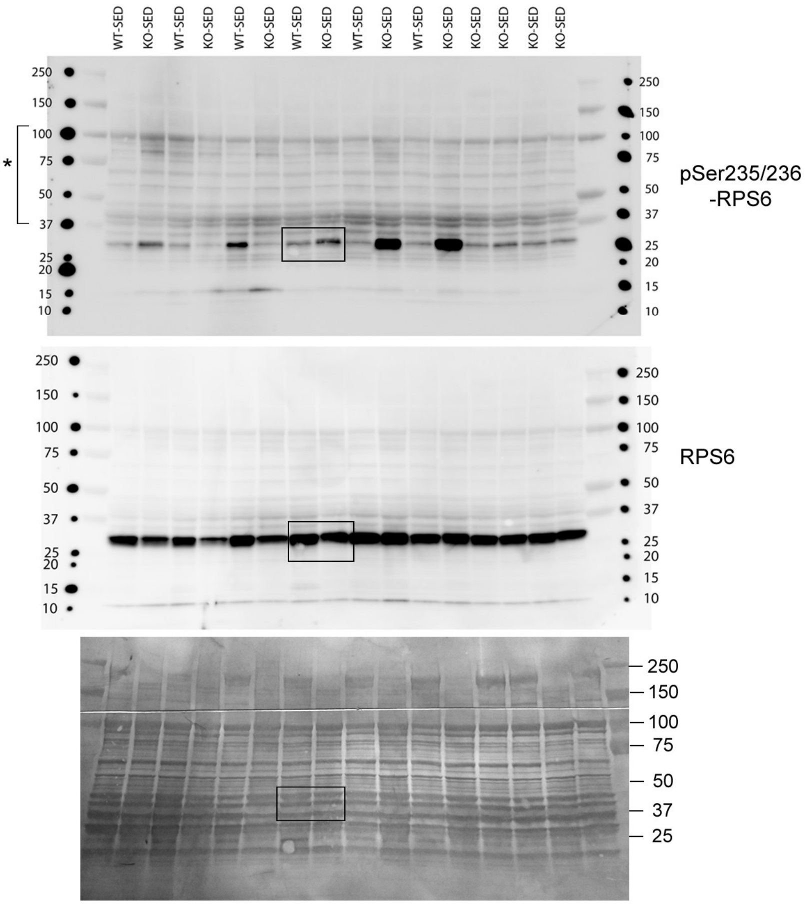

Coomassie

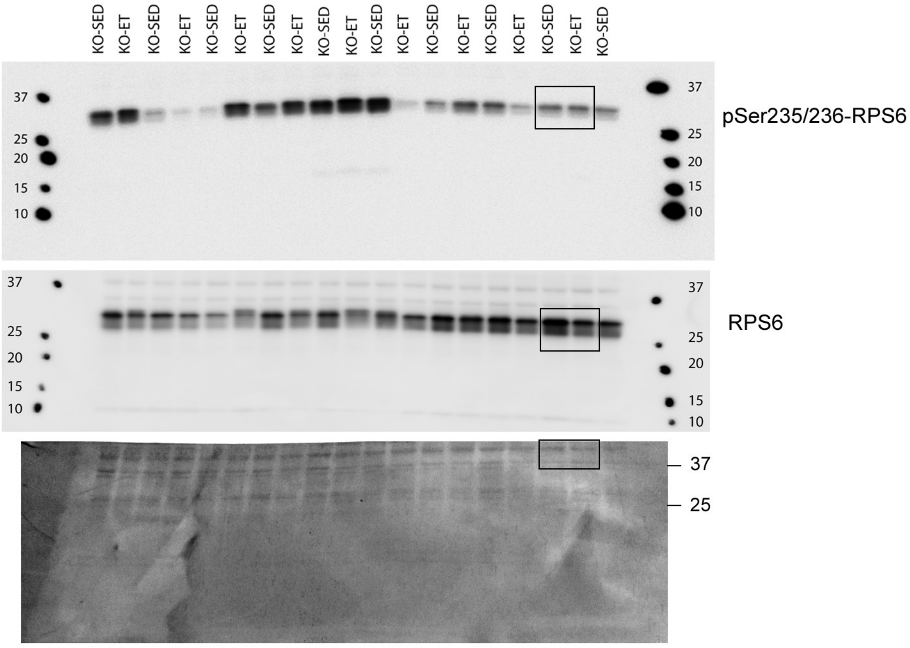

Coomassie

* Unspecific binding

Fig. 5A (cleaved Notch)

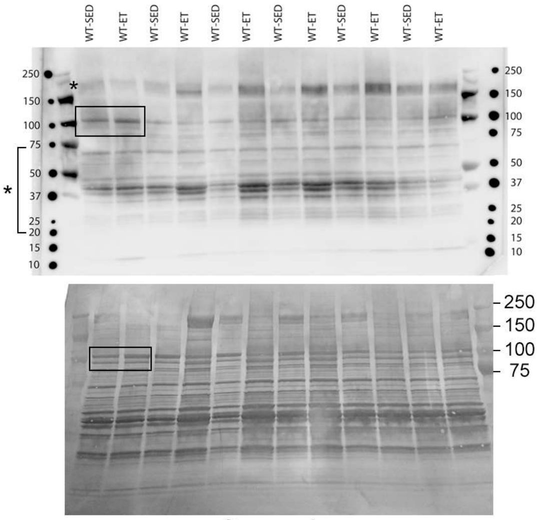

Coomassie

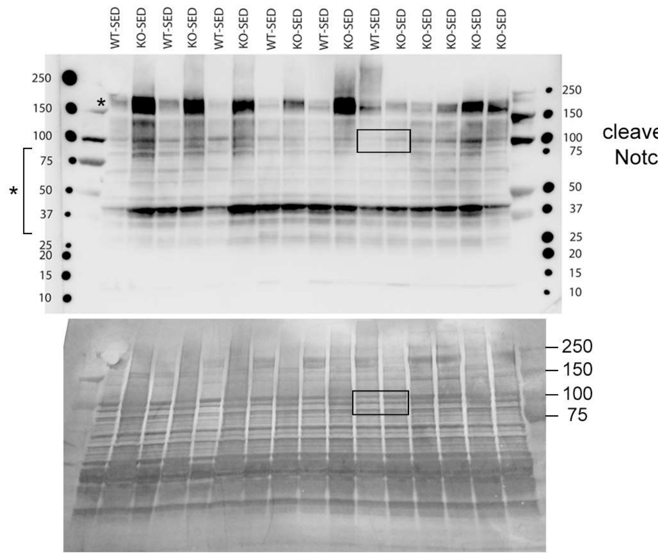

Coomassie

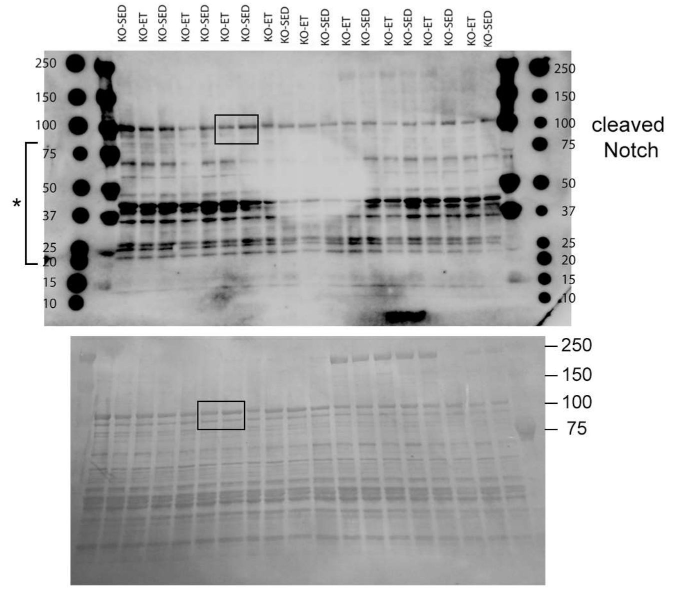

Coomassie

KO-SED and KO-ET: The boxed samples are flipped horizontally in Fig. 5A

* Unspecific binding

Fig. 5B (HES-1)

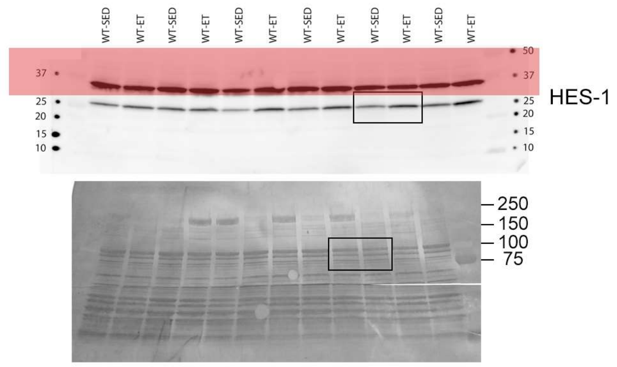

Coomassie

Strong bands in red box are background from another antibody

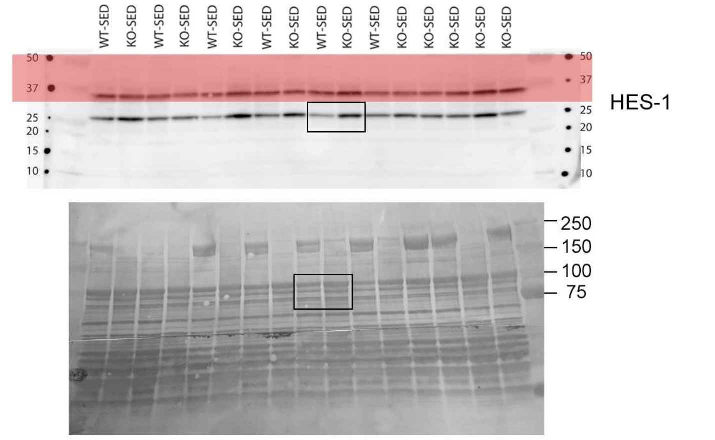

Coomassie

Strong bands in red box are background from another antibody

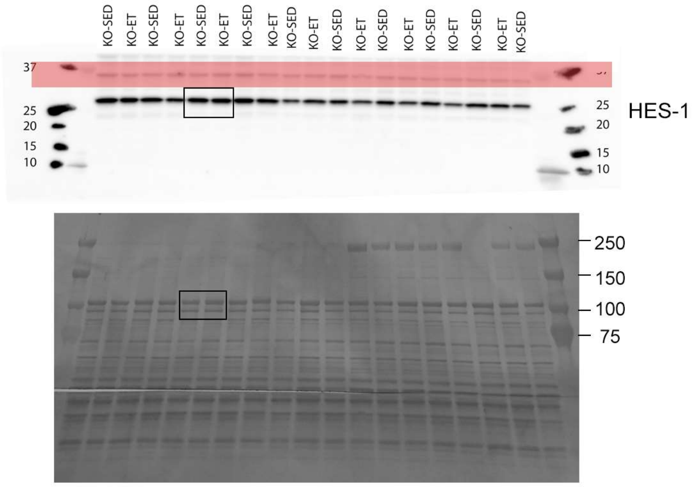

Coomassie

Strong bands in red box are background from another antibody

* Unspecific binding

Fig. 5C (TRPC7)

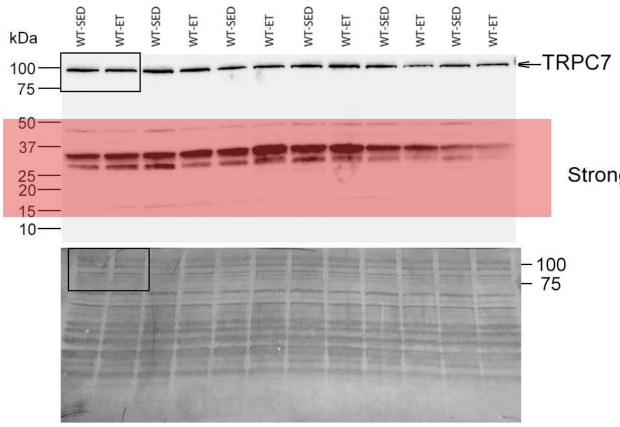

Strong bands in red box are background from another antibody

Coomassie

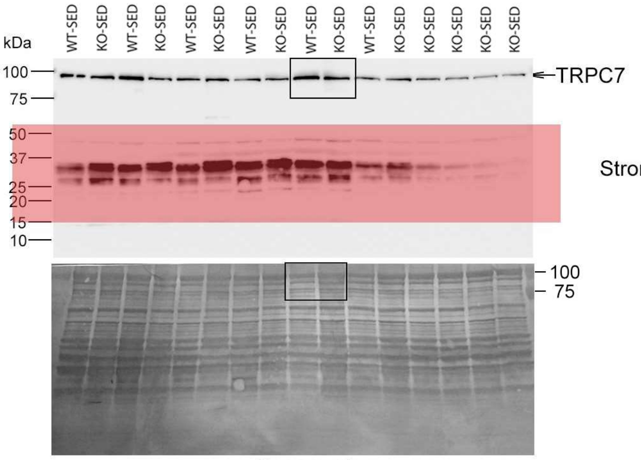

Strong bands in red box are background from another antibody

Coomassie

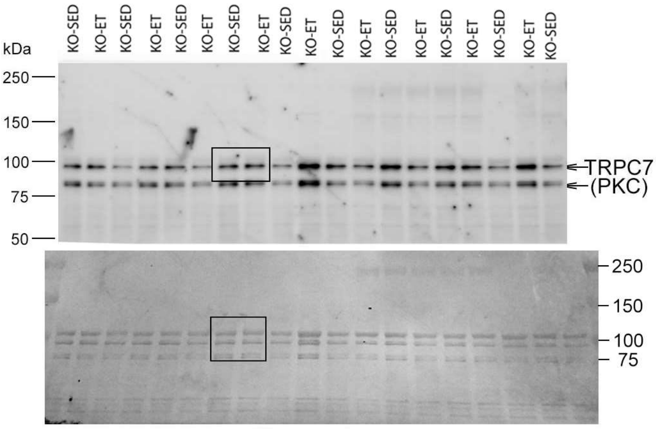

Coomassie

The lower blot was first probe with
PKC and thereafter stripped and repobed with TRPC7

Suppl. Fig. 1B (PAX-7)

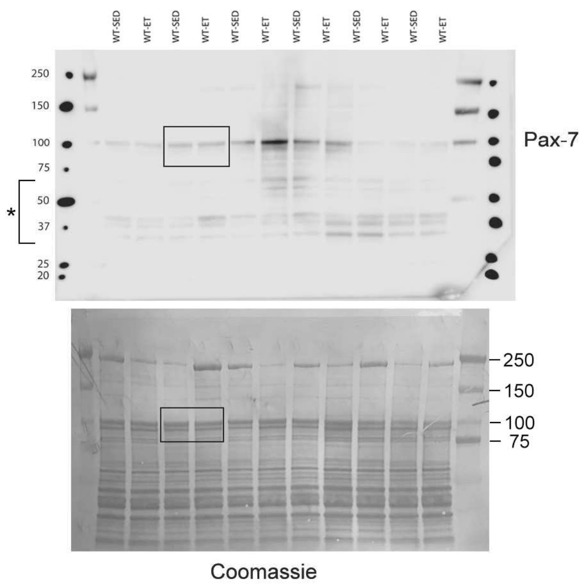

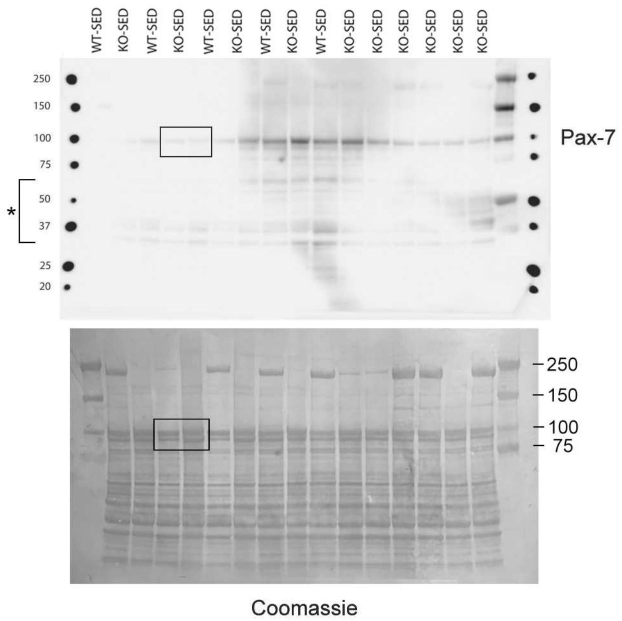

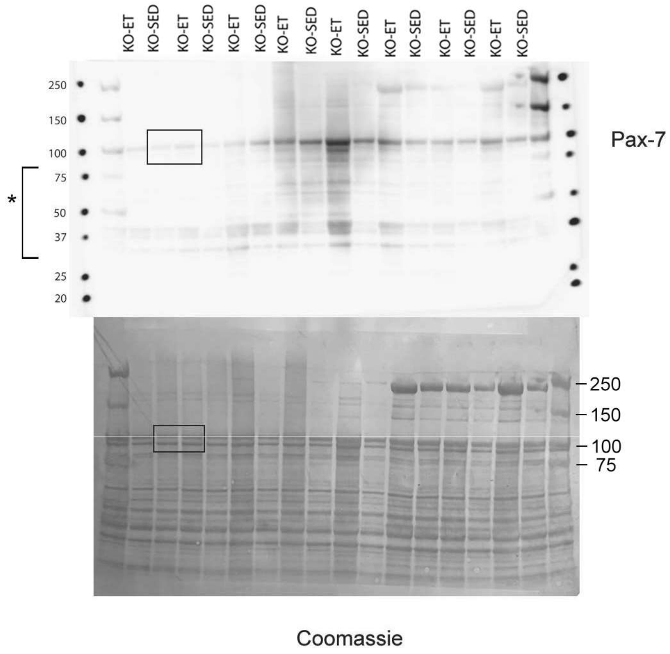

* Unspecific binding

Suppl. Fig. 2A (pThr308-Akt)

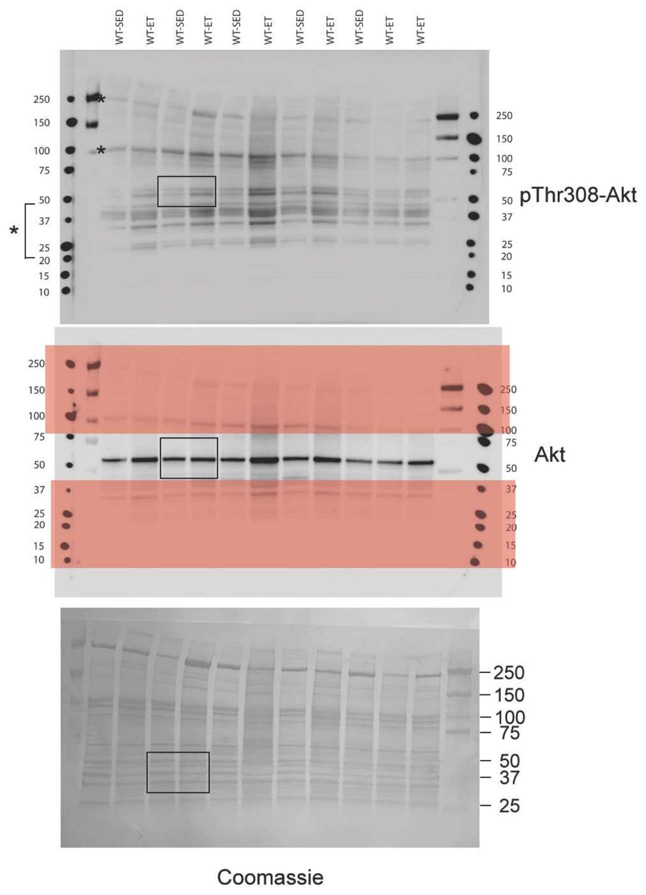

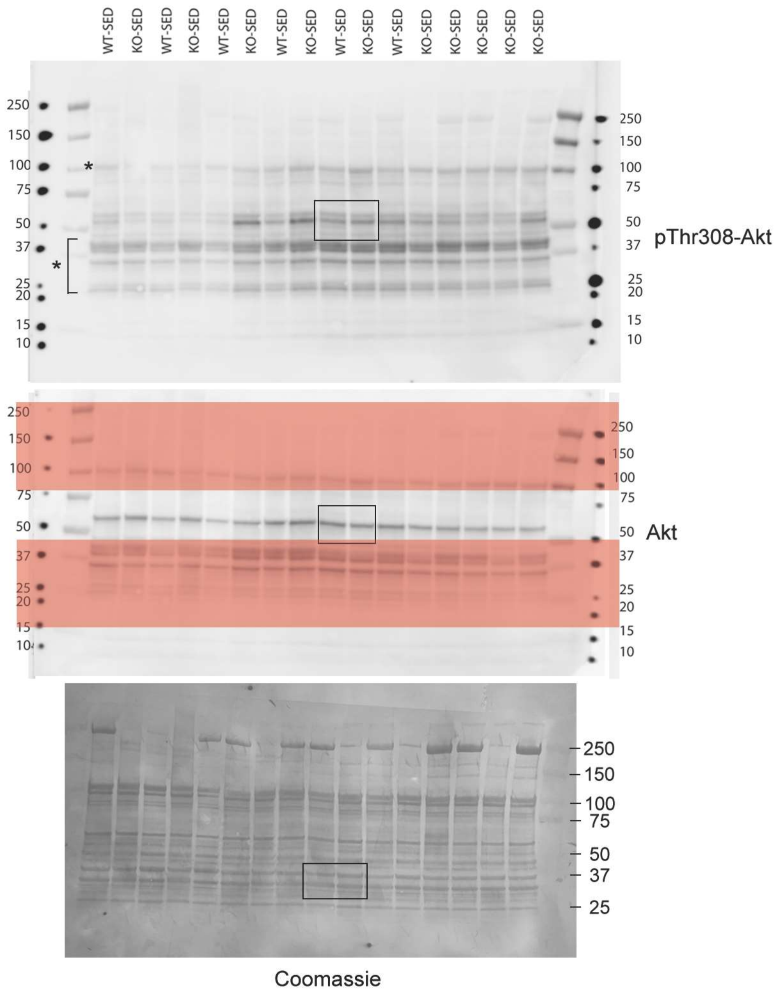

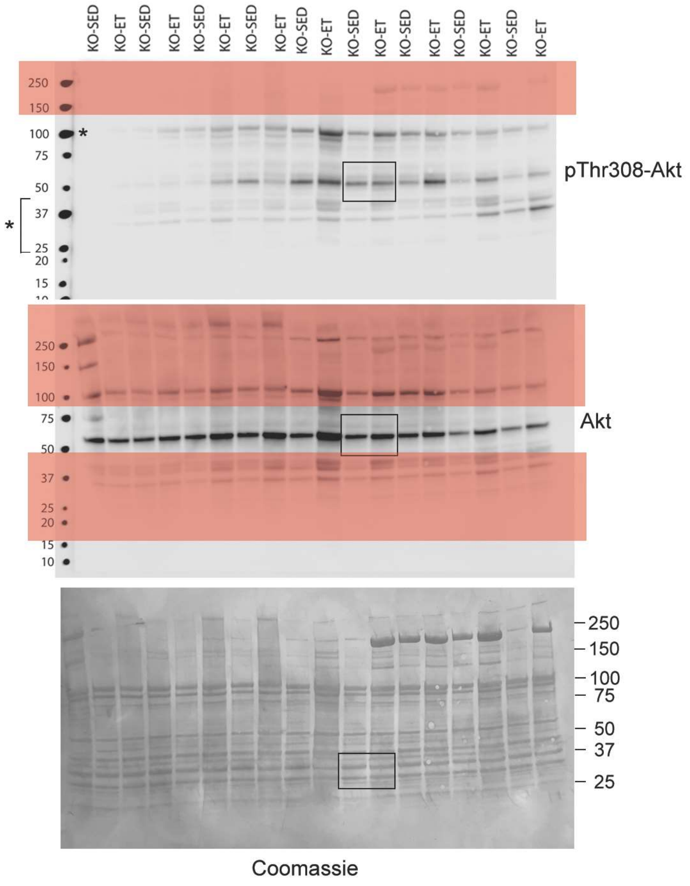

* Unspecific binding

Strong bands in red boxes are background from another antibody.

Suppl. Fig. 2B (pSer240/244-RPS6)

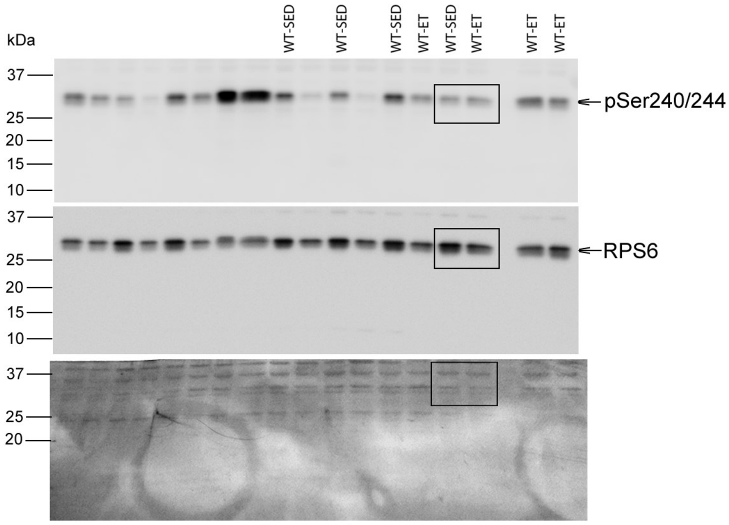

Coomassie

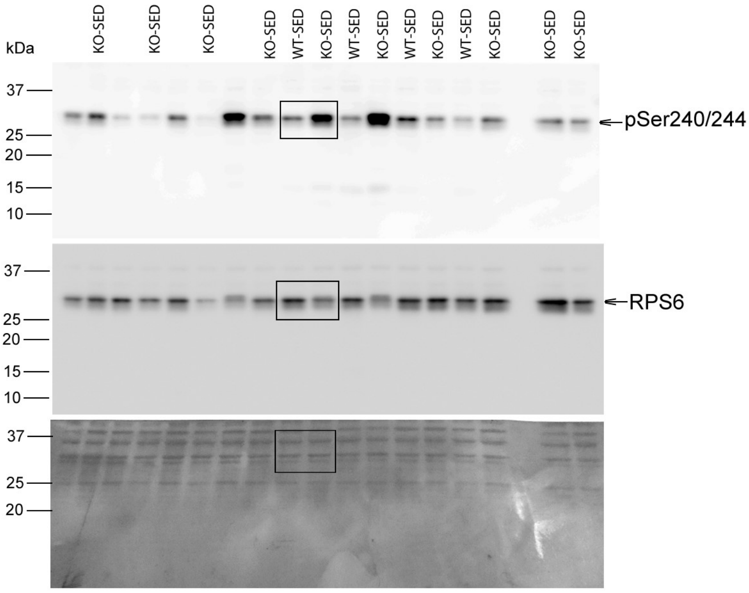

Coomassie

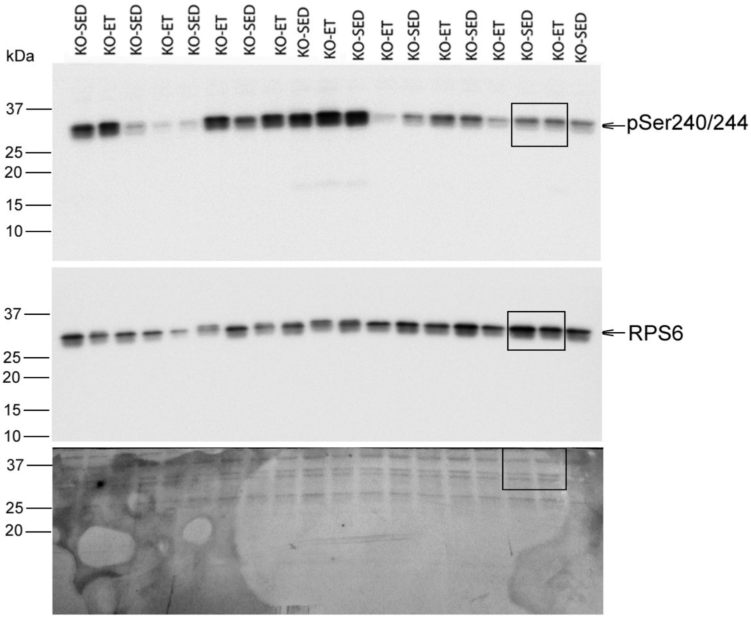

Coomassie

Non-littermates are removed

Suppl. Fig 2C (Dvl)

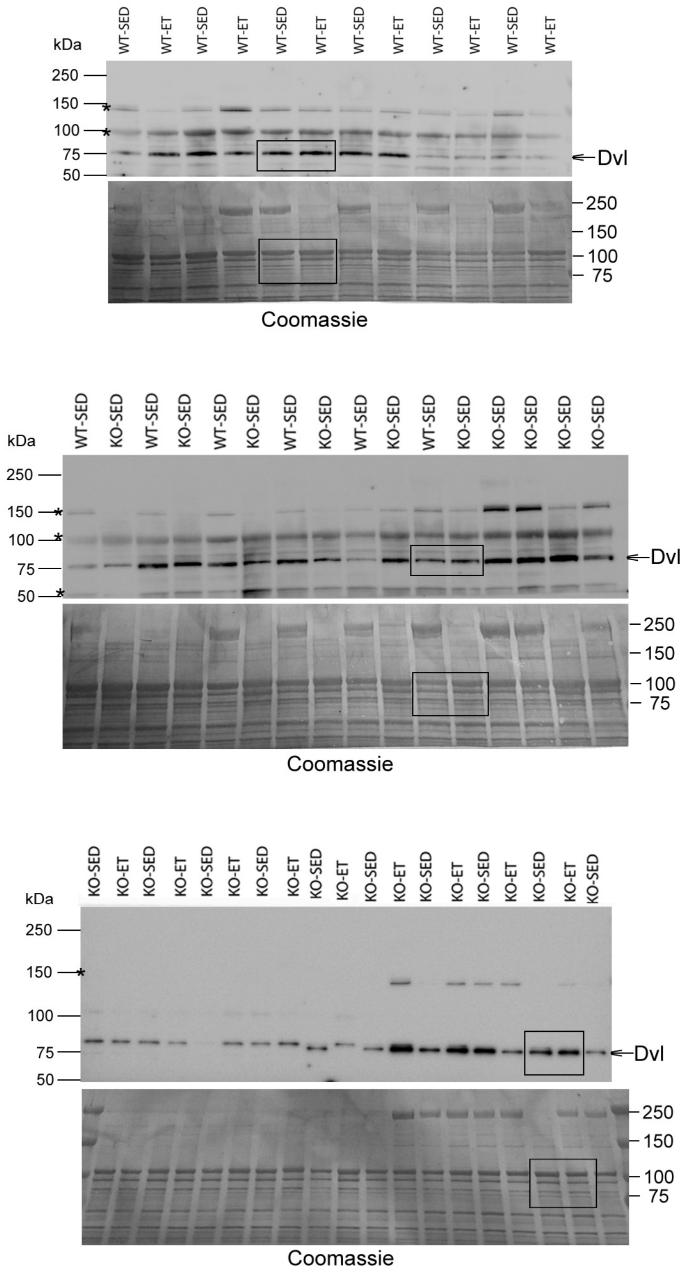

* Unspecific binding

Suppl. Fig 2D (beta-catenin)

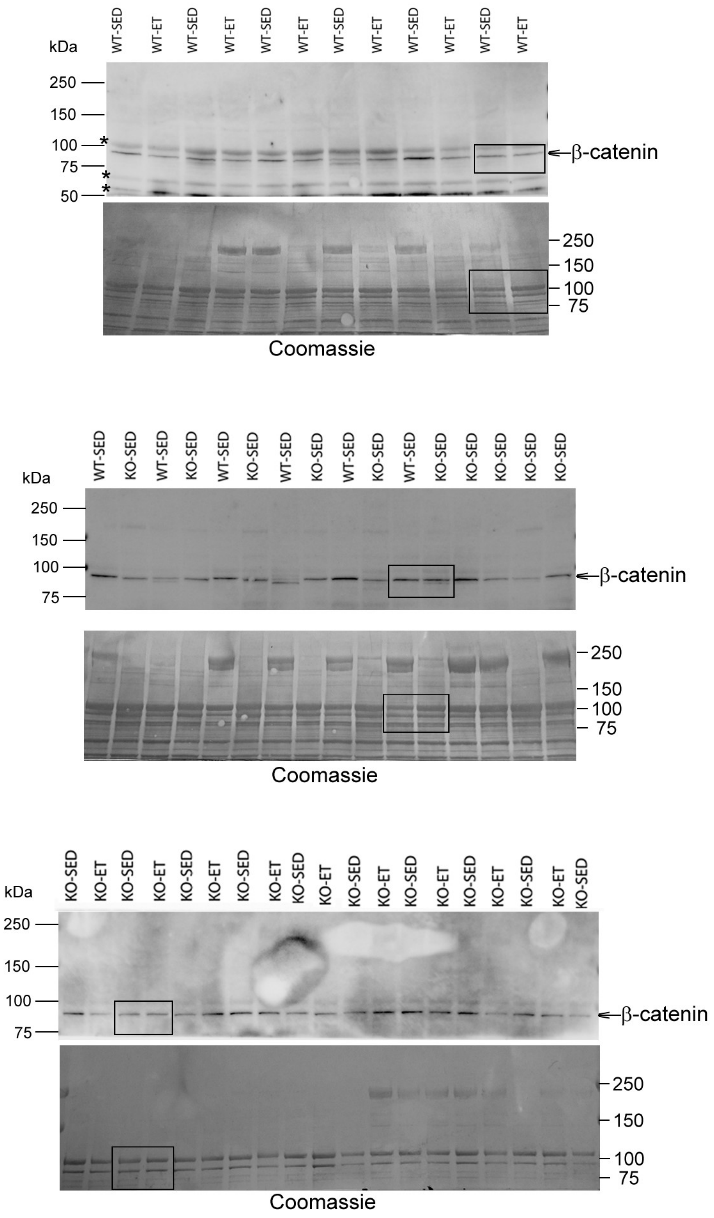

* Unspecific binding

Suppl. Fig. 2E (Frizzled-7)

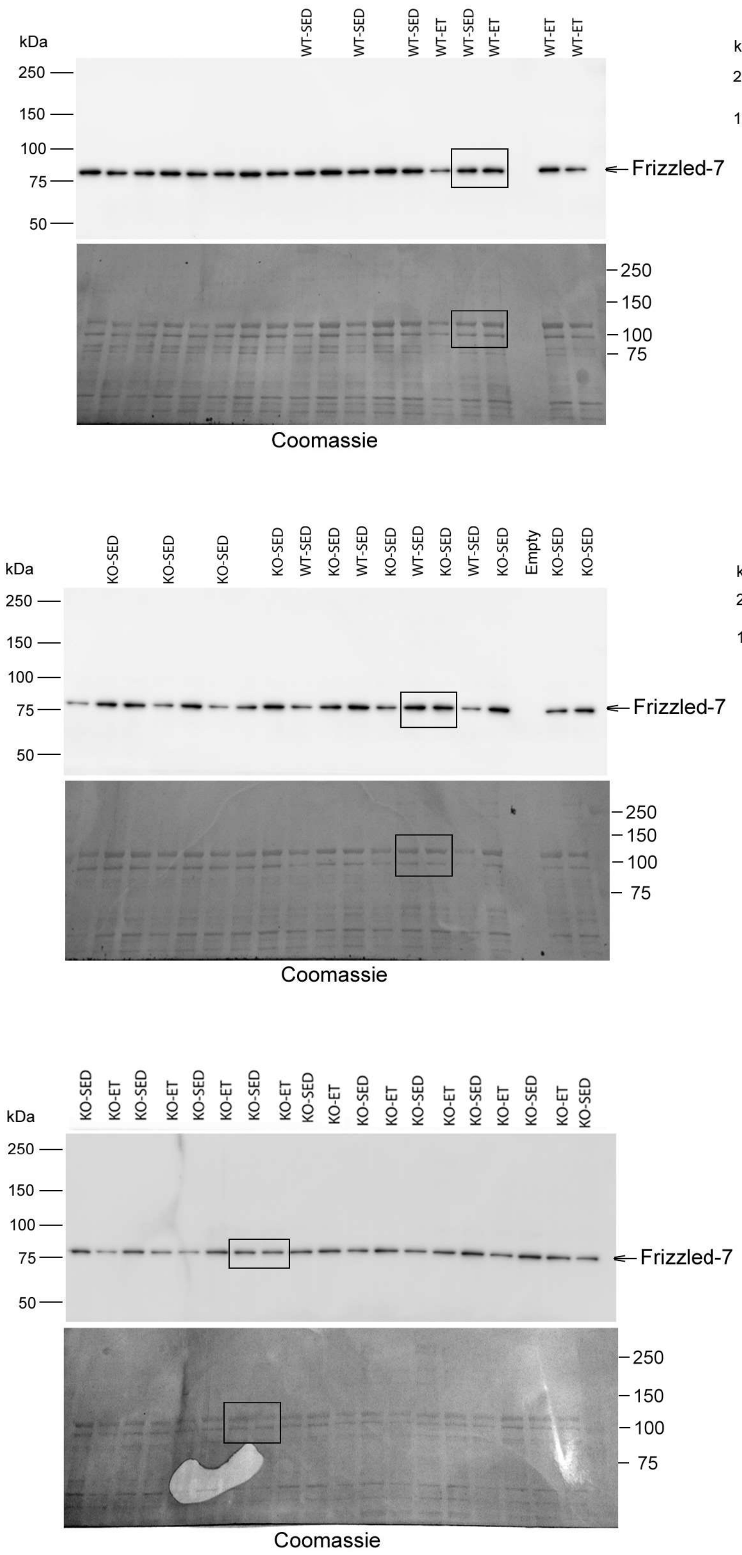

Non-littermates are removed

Suppl. Fig. 2F (LRP6)

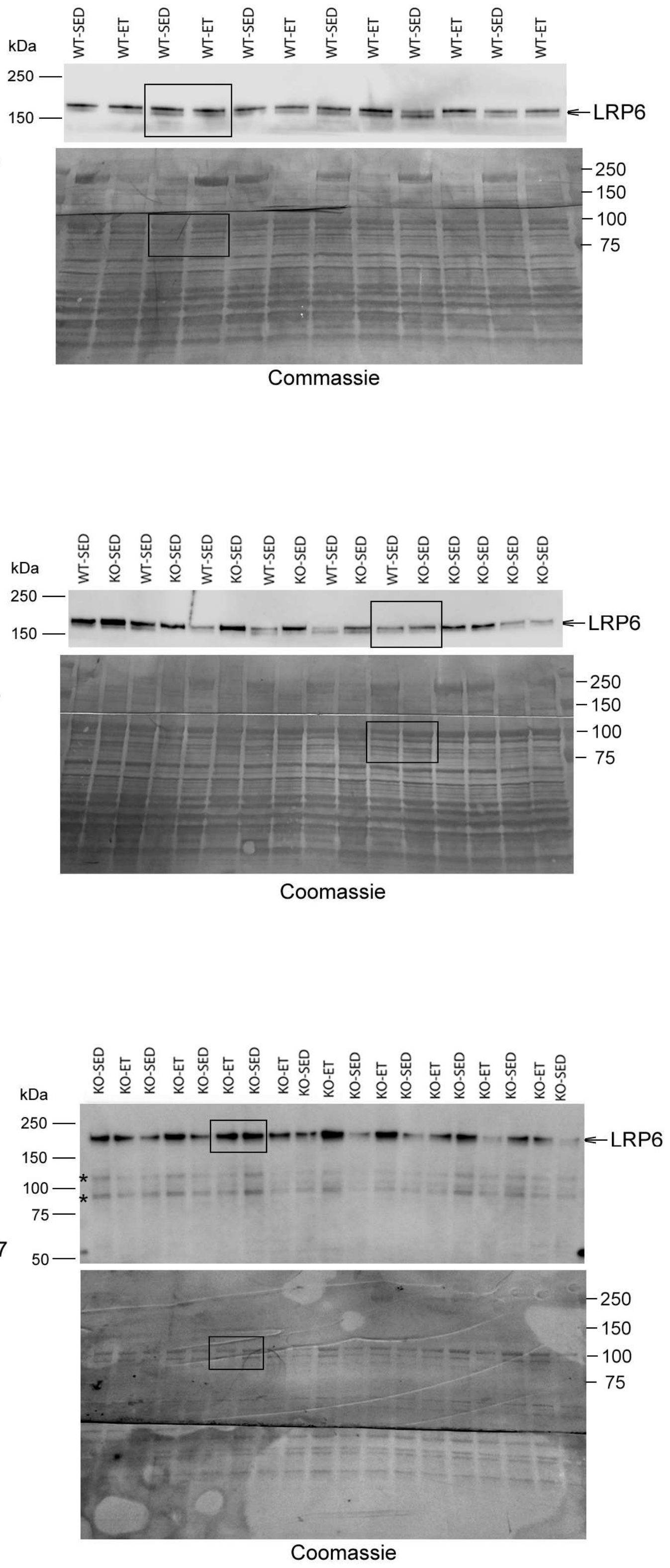

KO-SED vs KO-ET: The boxed samples are flipped horizontally in Suppl. Fig. 1F

* Unspecific binding

Suppl. Fig 2G (Wnt4)

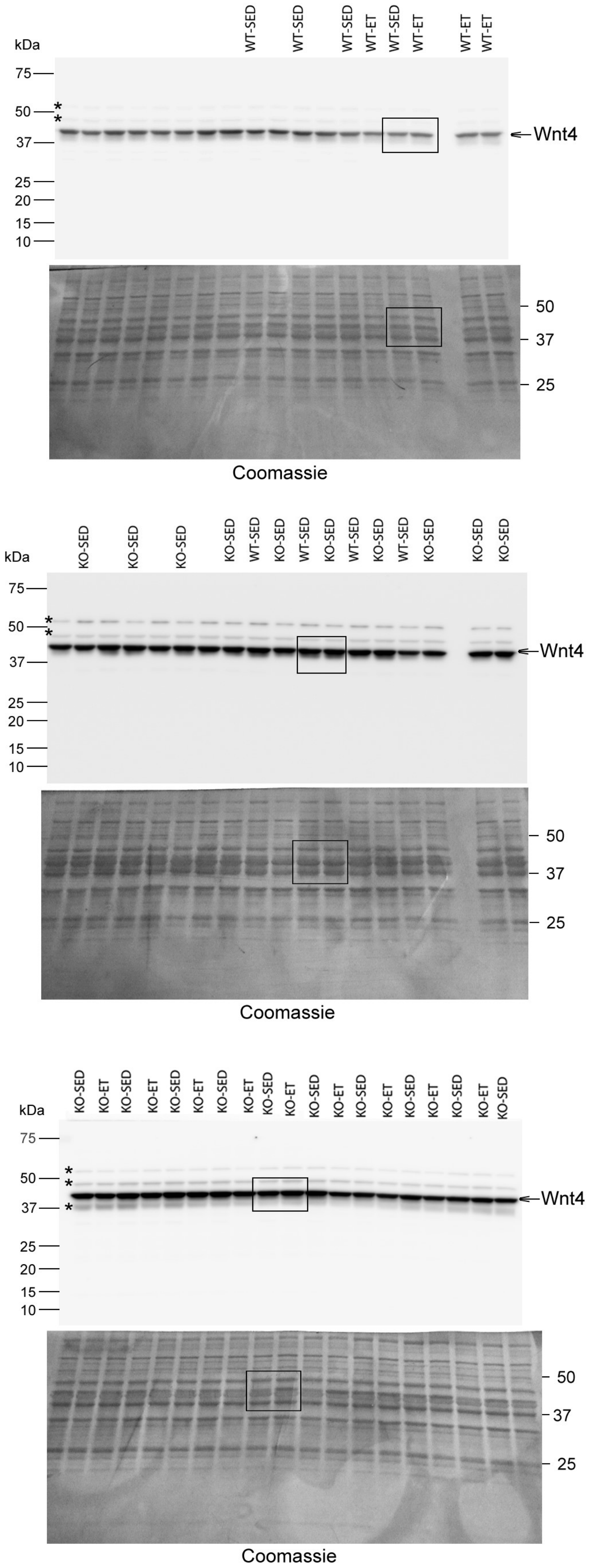

Suppl. Fig. 2H (PKC)

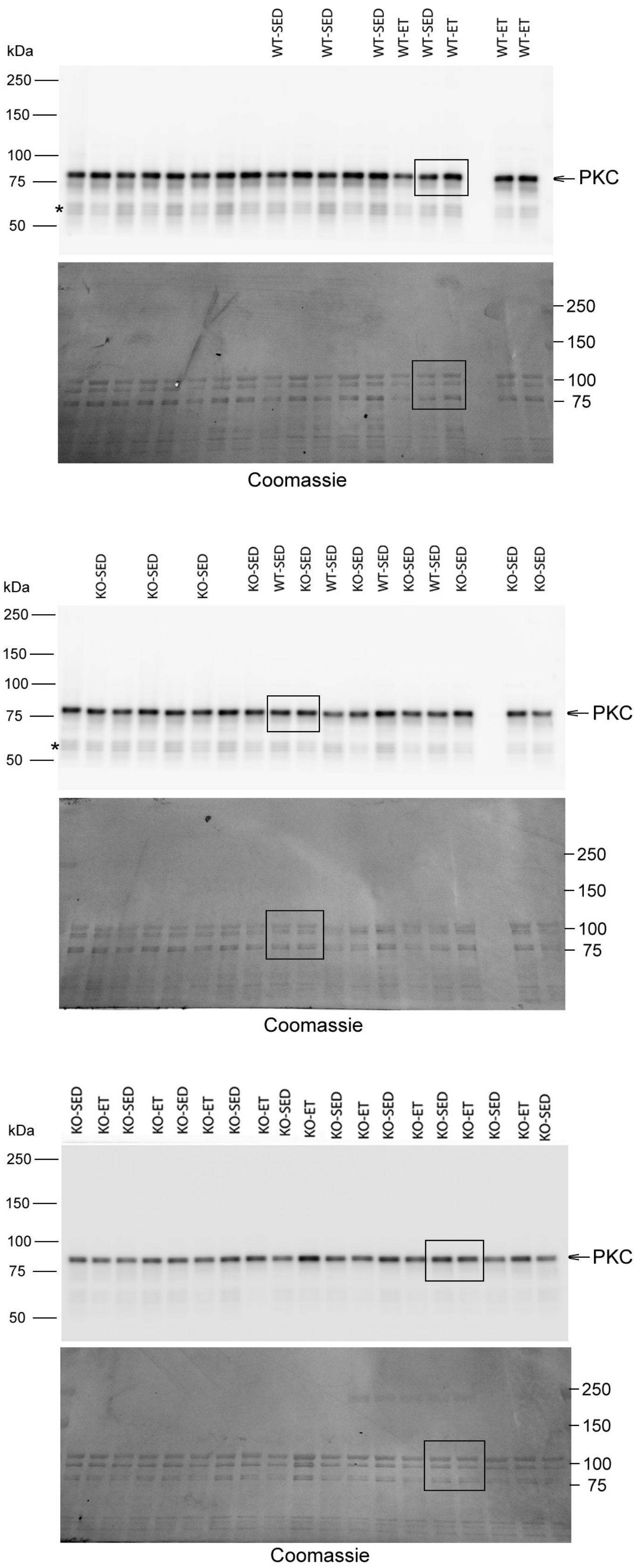

Non-littermates are removed

* Unspecific binding

Suppl. Fig 2I (RCAN4.1)

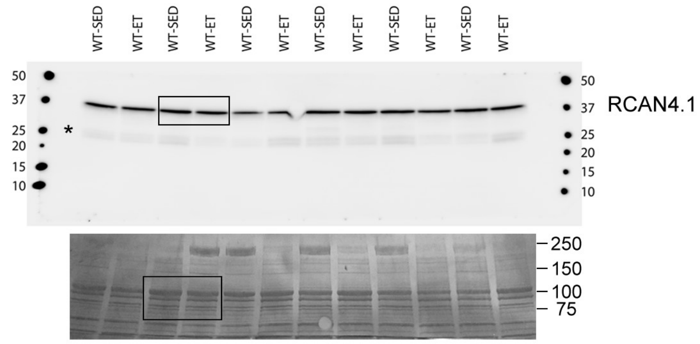

Coomassie

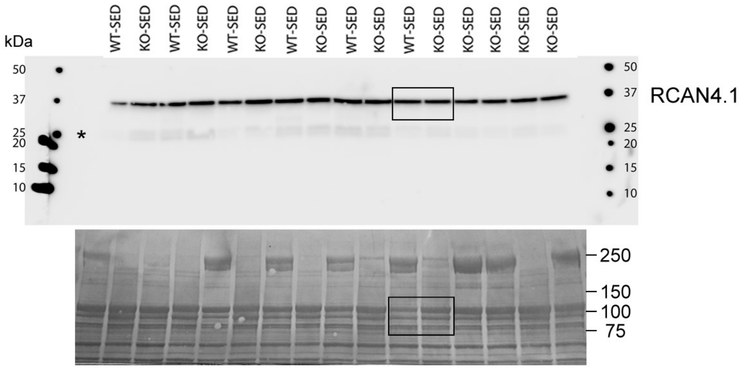

Coomassie

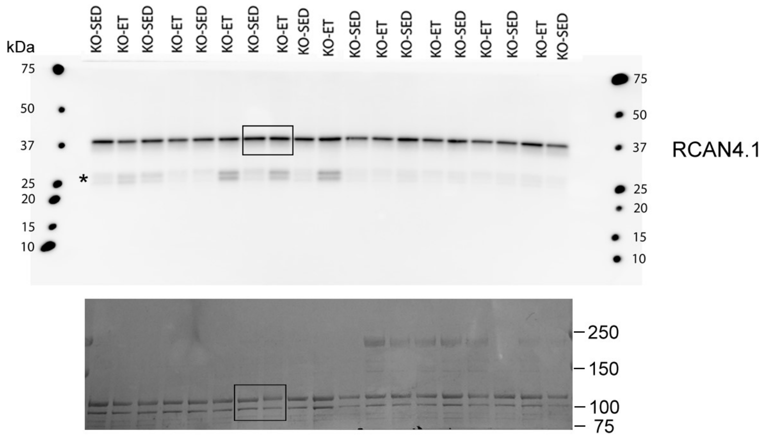

Coomassie

* Unspecific binding
