# Supplementary figures and images for: Syndecan-4–/– Mice Have Smaller Muscle Fibers, Increased Akt/mTOR/S6K1 and Notch/HES-1 Pathways, and Alterations in Extracellular Matrix Components
Source: Front Cell Dev Biol. 2020 Jul 31;8:730. doi: 10.3389/fcell.2020.00730 (PMC7411008; doi:10.3389/fcell.2020.00730)

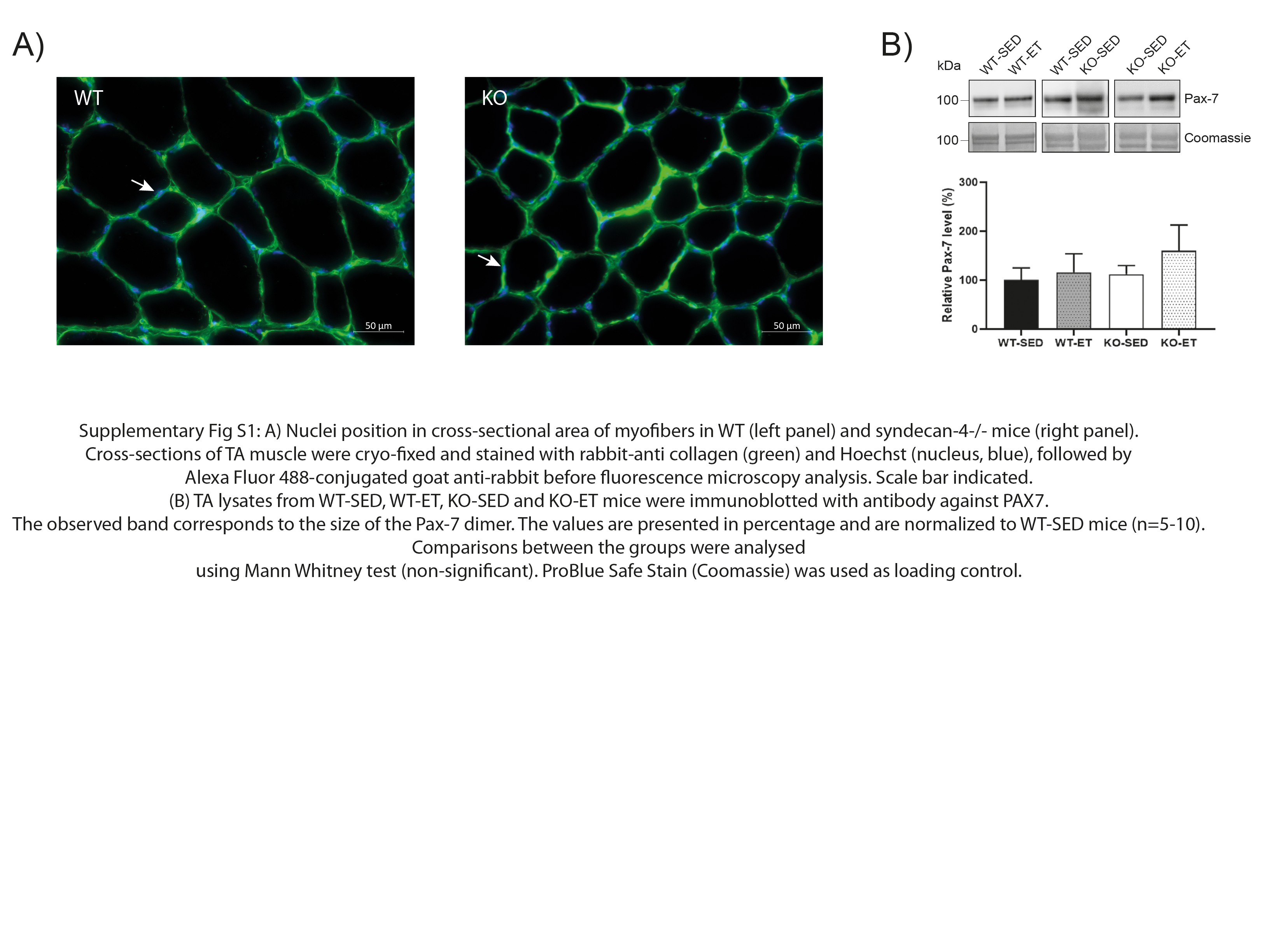

Supplement: Supplementary file 2 [file Image_1.TIF]

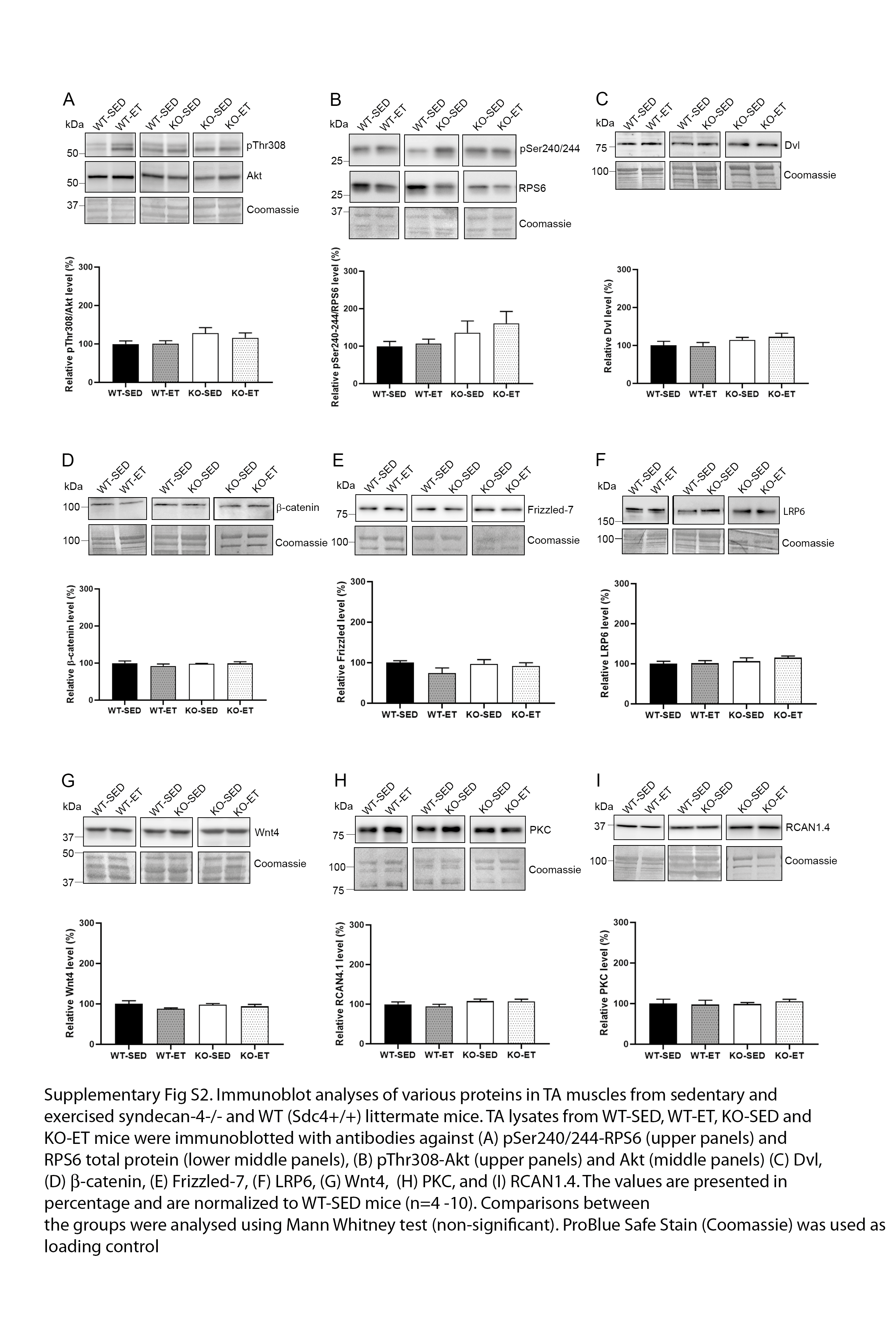

Supplement: Supplementary file 3 [file Image_2.TIF]
